# Enzymatic Deglycosylation and Lipophilization of Soy Glycosides into Value-Added Compounds for Food and Cosmetic Applications

**DOI:** 10.1021/acsomega.4c11325

**Published:** 2025-03-20

**Authors:** Matteo Corti, Francesca Annunziata, Agostina Colacicco, Lucia Tamborini, Francesco Molinari, Martina Letizia Contente, Andrea Pinto

**Affiliations:** † Department of Food Environmental and Nutritional Sciences (DeFENS), University of Milan, via Celoria 2, 20133 Milano, Italy; ‡ Department of Pharmaceutical Sciences (DISFARM), University of Milan, via Mangiagalli 25, 20133 Milano, Italy

## Abstract

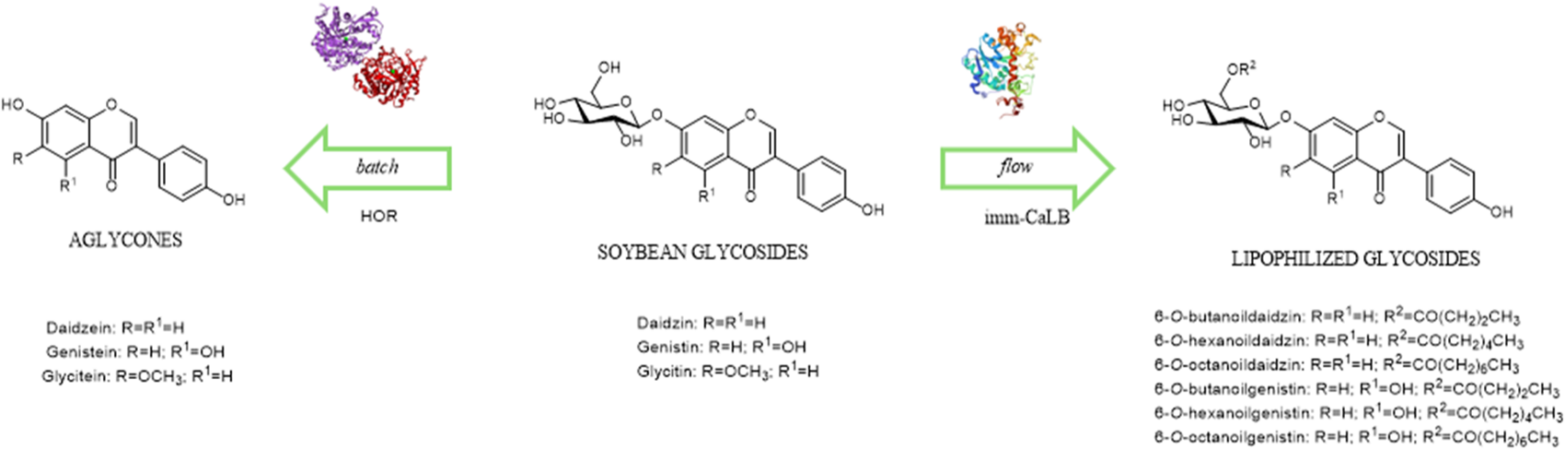

Soybean is one of the most important crops worldwide, being placed ninth on the chart of the most cultivated species. Its high level of production correlates with a huge amount of waste produced. These residues could be of great interest due to the presence of high-value-added molecules, including some glycosides (i.e., daidzin, genistin, glycitin) widely studied for their potent antioxidant properties. Due to their low bioavailability and limited solubility in lipidic media, lipophilization strategies have recently gained momentum to improve daidzin, genistin, and glycitin applications as multifunctional additives in the food, pharmaceutical, and cosmetic sectors. In this context, starting from soybean glycosides, we followed two parallel approaches, i.e., hydrolysis to obtain the corresponding aglycones possessing a better pharmacokinetic profile and esterification of the sugar primary alcohol with short-chain fatty acids. First, homemade extremophilic glycosidase (HOR) from *Halothermothrix orenii* has been employed for the preparation of aglycones (molar conversion 96–99%) in both water and biphasic media (water/2,2,5,5-tetramethyloxolane 1:1). Subsequently, lipophilization reactions with butanoic, hexanoic, and octanoic acids have been carried out using commercially available immobilized lipase B from *Candida antarctica* (CaLB) under flow conditions to produce modified glycosides with better physicochemical properties to be implemented in cosmetic preparations. Noteworthily, compared to the batch methodology, compound **1** (6-*O*-octanoildaidzin) was obtained with a drastic reduction in reaction time (30 min vs 18 h) and a consequent 9-fold increase in specific reaction rates (0.15 vs 0.017 μmol/(min·g)).

## 1 Introduction

*Glycine max* (L.) Merr., commonly known as soybean, is a leguminous plant in the family Fabaceae (Leguminosae) native to East Asia. It is known not only as a food legume with a high content in vegetable proteins but also as the most cultivated and used oilseed crop globally.^1,2^ The world soybean production reached an average annual production of 337 million tons in 2019/2020, resulting in the ninth most produced commodity worldwide between 1994 and 2022.^3^ One of the key factors contributing to the widespread production of soybeans is the fact they are a rich source of nutrients (i.e., proteins and fatty acids) and bioactive compounds, including isoflavones.^4^

Isoflavones are a class of estrogen-like nonsteroid substances found in plants, effective in preventing cancer, arteriosclerosis, osteoporosis, menopausal symptoms, and obesity.^5,6^ Isoflavones are also known for their antimicrobial and antioxidant properties, which make them suitable multifunctional additives for pharmaceutical, nutraceutical, and cosmetic preparations.^7^ However, like other flavonoids, isoflavones suffer from chemical instability, low solubility in lipid-rich media, and poor oral bioavailability. Various strategies have been investigated to overcome these shortcomings, modifying the parent compounds chemically or enzymatically.^8,9^ The poor chemical stability and the low solubility in lipidic media could be overcome through lipophilization strategies where one or more polar groups of the original compound are replaced with nonpolar moieties while maintaining their original biological characteristics.^10^ Aglycones and glycosides show a different bioavailability; in fact, the glycosidic forms have longer absorbance times (daidzin and genistin are absorbed, respectively, in 9 and 9.3 h), while their corresponding aglycone forms require less time (absorbed in 5.2 and 6.6 h, respectively).^11^

**Figure 1:**
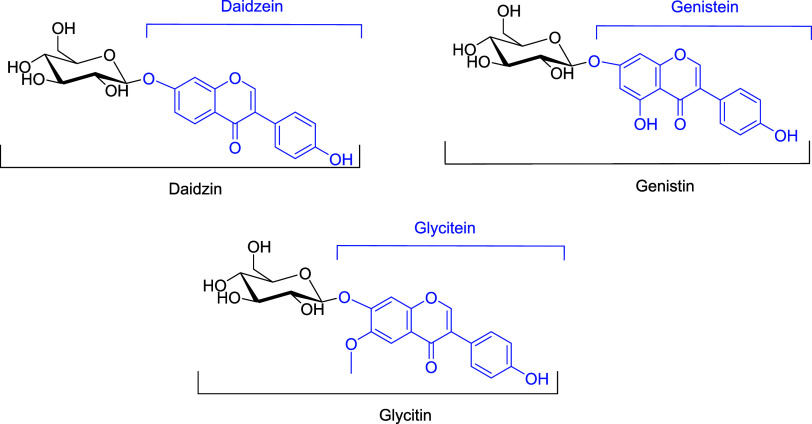
Chemical structures of daidzin, genistin, and glycitin and their corresponding aglycones daidzein, genistein, and glycitein (in blue).

Between 2018 and 2019, the total mass of soybean agricultural waste produced was 398 million tons. Considering that the average isoflavone content reported in leaf, branch, and pod waste is 1.2 kg of isoflavones/ton of waste, it has been estimated that 550,000 tons of isoflavones had been misspent.^3^ Together with processing waste (i.e., okara, soymilk, soy flour, etc.), soybean residues could be employed to generate high-value-added compounds as food or nutraceutical ingredients.^12^ The most common isoflavones in soybean and its residues are daidzein, genistein, and glycitein, which occur as aglycones and glycosides (i.e., daidzin, genistin, and glycitin) (Figure 1).^13^

As mentioned above, both chemical and enzymatic approaches have been explored to modify isoflavones. In recent years, enzymatic reactions are capturing attention, thanks to their advantageous features such as chemo-, regio-, and stereoselectivity, which contribute to the development of very selective processes. Moreover, compared to a classical chemical approach, milder reaction conditions (i.e., temperature, pH, pressure, and reaction medium) are required,^14,15^ thus increasing the overall process sustainability.^16,17^ Therefore, the combination of biocatalyzed reactions and continuous-flow chemistry has emerged in the last decades as a convenient and efficient synthetic approach for sustainable and intensified processes.^18−21^

In this context, the present work aims at the valorization of the three mentioned isoflavone glycosides usually present in the soybean residues through two different biocatalytic approaches, which allow the obtainment of derivatives endowed with tailor-made physical and chemical properties (e.g., chemical and metabolic stability, hydrophilic–lipophilic balance).^22−24^ First, an extremophilic β-glycosidase from the halothermophilic bacterium *Halothermothrix orenii* (HOR) was employed to cleave glucose from the selected glycosides to obtain the corresponding aglycones characterized by increased lipophilicity and biological activity.^25,26^ This approach allows us to avoid chemical hydrolysis using strong concentrated inorganic acids.^27^ Then, the commercially available immobilized lipase B from *Candida antarctica* (CaLB) was utilized for the esterification of daidzin with different fatty acids in batch conditions, thus allowing to increase the lipophilicity without altering the antioxidant properties given by the phenols;^28−30^ the latter procedure was then transferred into a continuous-flow reactor, allowing faster reaction times and higher productivity. In this context, a biocatalytic approach allowed to selectively acylate the *O*-6-glucose position without the need of protection/deprotection steps usually required by chemical acylation.^31^

## 2 Materials and Methods

Commercially available reagents and cell growth media were purchased from Merck or Thermo Fisher Scientific. Organic solvents and chemical standards were purchased from Merck. Immobilized lipase B from *C. antarctica* (CaLB) and *Thermomyces lanuginosus* lipase (TLL) were purchased from Merck. NMR spectra were recorded on a Bruker Avance Neo 400 MHz spectrometer using the residual signal of the deuterated solvent as an internal standard. Chemical shifts (δ) are expressed in parts per million, and coupling constants (*J*) are expressed in Hertz (Hz). Merck silica gel 60 F254 (aluminum foil) plates were used for analytical thin layer chromatography (TLC); column chromatography was performed on Merck silica gel (230–400 mesh). Compounds on the TLC plates were detected under UV light at 254 nm. Continuous-flow biotransformations were performed using Asia Flow Chemistry Syringe pumps (Syrris) and the heating unit of the R4 flow reactor (Vaportec) equipped with an Omnifit glass column (6.6 mm i.d. × 100 mm length). The temperature sensor sits on the wall of the reactors. Pressure was controlled using back-pressure regulators. High-performance liquid chromatography (HPLC) analyses were carried out on a Merck-Hitachi LaChrom liquid chromatograph with an L-7200 autosampler, an L-7100 pump, and an L-7400 UV detector; column LiChroCART (250 mm × 4.6 mm × 5 μm); flow rate 1 mL/min; λ = 280 nm; mobile phase with gradient of water (A) and acetonitrile (B): 0–10 min, 100% A; 10–20 min, 80% A; 20–30 min, 70% A; 30–35 min, 40% A; 35–45 min, 0% A; 45–55 min, 90% A. Retention times: daidzin: 16.1 min, daidzein: 26.8 min, genistin: 18.2 min, genistein: 29.8 min, glycitin: 15.8 min, glycitein: 27.2 min, 6-*O*-butanoildaidzin: 26 min, 6-*O*-hexanoildaidzin: 28.9 min, 6-*O*-octanoildaidzin: 32.2 min, 6-*O*-butanoilgenisin: 28.8 min, 6-*O*-hexanoilgenistin: 31.2 min, and 6-*O*-octanoilgenistin: 33.5 min.

### 2.1 Expression and Purification of HOR

Protein expression and purification were carried out as previously reported by Delgado et al. The protein was purified with the AKTA start system (GE Healthcare). HOR was expressed with good yields (66 mg/L of culture) and analyzed by sodium dodecyl sulfate-polyacrylamide gel electrophoresis (SDS-PAGE) (Figure S1).

### 2.2 HOR Activity Assay

Activity assay was carried out as described by Delgado and colleagues.^32^ The specific activity (U/mg) was expressed as μmol of product formed per minute per milligram of enzyme. The HOR final specific activity of HOR was 11 U/mg.

### 2.3 Biotransformation of Glycosides to Aglycones

Batch reactions using HOR were performed in 10 mL screw-cap tubes; 5 mL of reaction mixtures contained 5 mg/mL glycoside and 0.5 mg/mL enzyme under two conditions: *N*-(2-hydroxyethyl)piperazine-*N*′-ethanesulfonic acid (HEPES) buffer 50 mM, pH 7.4, with 10% dimethyl sulfoxide (DMSO) and biphasic system buffer/2,2,5,5-tetramethyloxolane (TMO) 1:1. Reactions were left under magnetic stirring at 28 °C and monitored through TLC (dichloromethane (DCM)/MeOH 9:1, under UV light at 245 nm) and HPLC (Figures S3.1–S3.3). Complete conversions into daidzein, genistein, and glycitein were observed, respectively, in 60, 15, and 240 min. While the biphasic medium allowed for a direct separation of the reaction products from the catalyst, an extraction with EtOAc (3 × 10 mL) was necessary for buffer-based biotransformation. In both cases, the organic phase was collected, dried over Na_2_SO_4_, filtered, and evaporated under reduced pressure. The crude extract was purified by silica gel column chromatography (DCM/MeOH 9:1) and the products were assessed by ^1^H NMR and ^13^C NMR (Figures S4.1–S4.6).

### 2.4 Lipophilization Reaction in Batch

The model batch reaction was performed on 8 mL volume with 16 mg of daidzin (2 mg/mL), 30 μL of octanoic acid, and 100 mg of CaLB and acetone as the solvent, in the presence of 4 Å molecular sieves. The reaction was performed at 50 °C for 18 h under magnetic stirring. The reaction was monitored through TLC (DCM/MeOH 9:1, under UV light at 245 nm) and HPLC (Figure S3.4). At the end, the reaction mixture was filtered, the solvent was evaporated under reduced pressure, and the crude product was purified by silica gel column chromatography (DCM/MeOH 9:1). The obtained product was assessed by ^1^H NMR and ^13^C NMR.

### 2.5 Flow Lipophilization Reactions

A glass column (i.d.: 6.6 mm) was packed with a previously prepared mixture of CaLB (500 mg) and powder molecular sieves 4 Å (500 mg) (PBR volume: 2.4 mL). A solution of daidzin (2 mg/mL) and the selected organic acid (1:5 molar ratio) in acetone (15 mL) was made to flow into the reactor column and kept at 70 °C. A constant pressurization of 3 bar was assured by the use of a back-pressure regulator. The total flow rate was 0.08 mL/min (residence time: 30 min). The reactions were monitored through TLC (DCM/MeOH 9:1, under UV light at 245 nm) and HPLC (Figures S3.5–S3.16). The organic solvent was collected and evaporated under reduced pressure, and the crude product was purified by silica gel column chromatography (DCM/MeOH 9:1). The obtained product was assessed by ^1^H NMR and ^13^C NMR (Figures S4.7–S4.15).

### 2.6 Compound Characterization

#### 2.6.1 Daidzein

^1^H NMR (400 MHz, DMSO-*d*_6_): δ 8.28 (s, 1H), 7.96 (d, *J* = 8.80 Hz, 1H), 7.38 (d, *J* = 8.60 Hz, 2H), 6.93 (d, *J* = 8.80, 2.20 Hz, 1H), 6.86 (d, *J* = 2.20 Hz, 2H), 6.84 (d, *J* = 8.60 Hz, 2H); ^13^C NMR (100 MHz, DMSO-*d*_6_): δ 175.2, 163.0, 157.9, 157.6, 153.2, 130.5 (2C), 127.7, 123.9, 123.0, 117.1, 115.6, 115.4 (2C), 102.6.

#### 2.6.2 Genistein

^1^H NMR (400 MHz, DMSO-*d*_6_): δ 8.31 (s, 1H), 7.37 (d, *J* = 8.60 Hz, 2H), 6.82 (d, *J* = 8.60 Hz, 2H), 6.38 (d, *J* = 2.10 Hz, 1H), 6.22 (d, *J* = 2.10 Hz, 1H); ^13^C NMR (100 MHz, DMSO-*d*_6_): δ 180.7, 164.8, 162.5, 158.0, 157.9, 154.4, 130.6 (2C), 122.7, 121.7, 115.5 (2C), 104.9, 99.4, 94.1.

#### 2.6.3 Glycitein

^1^H NMR (400 MHz, DMSO-*d*_6_): δ 8.27 (s, 1H), 7.43 (s, 1H), 7.38 (d, *J* = 8.60 Hz, 2H), 6.94 (s, 1H), 6.81 (d, *J* = 8.60 Hz, 2H), 3.88 (s, 3H); ^13^C NMR (100 MHz, DMSO-*d*_6_): δ 174.8, 157.6, 153.5, 152.9, 152.2, 147.4, 130.5 (2C), 123.5, 123.4, 116.6, 115.4 (2C), 105.2, 103.28, 56.3.

#### 2.6.4 6-*O*-Butanoildaidzin

^1^H NMR (400 MHz, acetone-*d*_6_): δ 8.23 (s, 1H), 8.14 (d, *J* = 8.80 Hz, 1H), 7.49 (d, *J* = 8.70 Hz, 2H), 7.22 (d, *J* = 2.30 Hz, 1H), 7.16 (d, *J* = 2.30; H), 6.91 (d, *J* = 8.70 Hz, 2H), 5.23 (d, *J* = 7.30 Hz, 1H), 4.52 (dd, *J* = 11.80, 2.10 Hz, 1H), 4.24 (dd, *J* = 11.90, 7.20 Hz, 1H), 3.91 (td, *J* = 9.50, 7.20, 2.10 Hz, 1H), 3.62 (q, *J* = 8.90 Hz, 2H), 3.48 (t, *J* = 9.60 Hz, 1H), 3.23 (s, 1H), 2.35 (t, *J* = 7.30 Hz, 2H), 1.62 (sestet, *J* = 7.20 Hz, 2H), 0.91 (t, *J* = 7.40 Hz, 3H); ^13^C NMR (100 MHz, acetone-*d*_6_): δ 174.8, 172.5, 161.7, 157.5, 157.4, 152.6, 130.2 (2C), 127.2, 124.6, 123.3, 119.4, 115.5, 115.0 (2C), 103.6, 100.5, 76.9, 74.4, 73.6, 70.4, 63.3, 35.6, 18.1, 13.0.

#### 2.6.5 6-*O*-Hexanoildaidzin

^1^H NMR (400 MHz, acetone-*d*_6_): δ 8.23 (s, 1H), 8.14 (d, *J* = 8.80 Hz, 1H), 7.49 (d, *J* = 8.70 Hz, 2H), 7.22 (d, *J* = 2.30 Hz, 1H), 7.16 (dd, *J* = 8.80, 2.30 Hz, 1H), 6.91 (d, *J* = 8.70 Hz, 2H), 5.24 (d, *J* = 7.30 Hz, 1H), 4.52 (dd, *J* = 11.80, 2.10, 11.60 Hz, 1H), 4.24 (dd, *J* = 11.80, 7.30 Hz, 1H), 3.91 (td, *J* = 9.50, 7.20, 2.10 Hz, 1H), 3.60 (q, *J* = 14.70, 7.5 Hz, 2H), 3.48 (t, *J* = 9.20 Hz, 1H), 2.36 (t, *J* = 7.40 Hz, 2H), 1.59 (q, *J* = 7.20, 14.40 Hz, 2H), 1.29 (q, *J* = 7.20, 3.20 Hz, 4H), 0.83 (t, *J* = 7.10 Hz, 3H); ^13^C NMR (100 MHz, acetone-*d*_6_): δ 174.8, 172.7, 161.7, 157.5, 157.4, 152.6, 130.2 (2C), 127.2, 124.6, 123.3, 119.4, 115.5, 115.0 (2C), 103.6, 100.4, 76.8, 74.3, 73.6, 70.4, 63.3, 33.6, 31.1, 24.4, 22.1, 13.3.

#### 2.6.6 6-*O*-Octanoildaidzin

^1^H NMR (400 MHz, acetone-*d*_6_): δ 8.23 (s; 1H), 8.14 (d, *J* = 8.80 Hz, 1H), 7.49 (d, *J* = 8.70 Hz, 2H), 7.22 (d, *J* = 2.30 Hz, 1H), 7.16 (dd, *J* = 8.80, 2.30 Hz, 1H), 6.91 (d, *J* = 8.70 Hz, 2H), 5.23 (d, *J* = 7.30 Hz, 2H), 4.50 (dd, *J* = 11.80, 2.10, 12.0 Hz, 1H), 3.90 (td; *J* = 9.60, 7.40, 2.10 Hz, 1H), 3.60 (q, *J* = 8.80 Hz, 2H), 3.47 (t, *J* = 8.80 Hz, 1H), 3.32 (s, 1H), 2.37 (t, *J* = 7.50 Hz, 2H), 1.59 (q, *J* = 14.90, 7.30 Hz, 4H), 1.22 (q, *J* = 3.20, 7.20 Hz, 6H), 0.83 (t, *J* = 6.90 Hz, 3H); ^13^C NMR (100 MHz, acetone-*d*_6_): δ 174.8, 172.7, 161.7, 157.5, 157.4, 152.6, 130.2 (2C), 127.2, 124.6, 123.3, 119.4, 115.2, 115.0 (2C), 103.5, 100.4, 76.9, 74.3, 73.6, 70.5, 63.3, 33.7, 31.5, 29.0 (2C), 24.8, 22.3, 13.4.

### 2.7 Calculation of Selected Properties of Tested Compounds

Selected properties of the compounds (i.e., molecular weight, clog *P*, and clog *S*) were calculated with OSIRIS DataWarrior (Table S1).

## 3 Results and Discussion

### 3.1 Aglycone Production

β-Glycosidases (or β-d-glucopyranoside glucohydrolases) are specific enzymes for the hydrolytic cleavage of terminal β-linked glucosyl residues. In literature, there are several examples of the hydrolysis of soy isoflavones catalyzed by β-glycosidases.^33−40^ We recently employed an extremophilic homemade β-glycosidase (HOR) for the hydrolysis of hesperidin (HES) and rutin (RT), two citrus rutinosyl flavonoids.^40^ In respect to other β-glycosidases, HOR is characterized by higher stability to both pH (active between 4.5 and 7) and temperature (with the optimum temperature between 65 and 70 °C).

Given the low solubility of daidzin, genistin, and glycine in HEPES buffer, it was necessary to select a suitable cosolvent to allow the complete solubilization of the substrates. Two different solvent systems have been compared: HEPES buffer (pH 7.4) with 10% DMSO and a biphasic system of HEPES buffer/TMO 1:1. TMO has recently been identified as a safer and greener alternative to toluene, tetrahydrofuran, and hydrocarbons.^41^ The transformation of daidzin into daidzein was used as a model reaction, dissolving 1.0 mg/mL enzyme and 5.0 mg/mL substrate in the above-mentioned solvent systems. Both the biotransformations showed complete conversion (>98% HPLC molar conversion) (Figure S3.1) after 60 min of reaction time at 28 °C. Therefore, the biphasic medium was chosen, with the purpose of avoiding the use of DMSO, which may interfere with the subsequent purification steps. Moreover, the biphasic system allowed the separation between the catalyst and the aglycone to be obtained, reducing the downstream processes. TMO has been recovered through evaporation and reused for several reaction cycles, thus reducing waste production of the proposed procedure.

The same conditions have also been adopted for the hydrolysis of genistin and glycitin. Both of them showed almost complete conversion after 15 and 240 min of reaction, respectively (Figures S3.2 and S3.3) (Table 1).

**Table 1:** Molar Conversions and Isolated Yields after HOR-Catalyzed Hydrolysis

substrate	product	time (min)	molar conversion (HPLC) (%)	isolated yield (%)
daidzin	daidzein	60	>98	>98
genistin	genistein	15	>99	80
glycitin	glycitein	240	96	50

The three products have been purified by column chromatography, giving a quantitative yield for daidzein, 80% for genistein, and 50% for glycitein. The low yield observed for glycitein could be explained with its very poor solubility in the organic solvent (Table 1).

### 3.2 Glycoside Lipophilization

**Figure 2:**
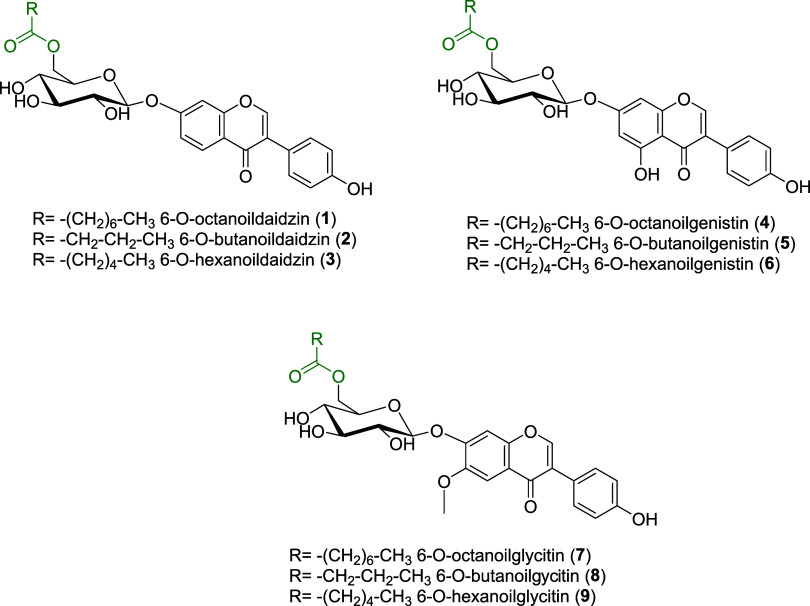
Chemical structure of the designed lipophilized esters.

The second goal of our work was the development of a biocatalyzed selective lipophilization of the flavonoid glycosides of interest through the esterification of the sugar primary alcohol with three selected fatty acids (Figure 2).

To increase the lipophilicity of the chosen compounds, leaving the phenol moiety untouched, closely related to the radical scavenger activity, a suitable biocatalyst to perform the acylation only on the glucose primary alcohol was selected. Lipases naturally catalyze the hydrolysis of the ester bond of tri-, di-, and monoglycerides into fatty acids and glycerol, at the interface of a biphasic system formed by an organic phase and a water medium.^42^ However, in organic solvents, lipases are able to catalyze condensation reactions as esterifications, amidations, and thio-esterifications.^42^ Lipases are the third largest group of commercialized enzymes, after proteases and glycosylases. Their wide use is connected to their stability in organic solvents, a wide variety of substrate acceptances, and selectivity, as well as cofactor independency. Therefore, two commercially immobilized lipases, CaLB and TLL, were selected and compared to perform the acylation using daidzin as a model substrate and acetone as a solvent. Since both catalysts showed similar reactivity at 50 °C, but only CaLB was able to work at 70 °C with an improvement in the conversion over time, this one was the biocatalyst of choice.

For the selection of the organic acids to be used for esterification, clog *P* of the acylated products was calculated using OSIRIS DataWarrior to obtain values similar to those of the aglycones without excessively increasing the molecular weight. Therefore, butanoic, hexanoic, and octanoic acids were chosen (Table S1).

Considering the reaction between daidzin and octanoic acid as a model reaction, the experimental parameters (i.e., solvent, concentration, amount of enzyme, and molar ratio) were varied evaluating the conversion after 24 h. Different green solvents were considered (i.e., 2-methyltetrahydrofuran, cyclopentyl methyl ether, dimethyl carbonate, *tert*-amyl alcohol, and acetone). Daidzin showed a higher solubilization at 5.0 mg/mL in only acetone and *tert*-amyl alcohol. Therefore, acetone was selected as the solvent for the biotransformation due to its lower boiling point and the resulting easy removal. Different concentrations of daidzin in acetone were tested (i.e., 2.0, 3.0, and 4.0 mg/mL) to assess the highest achievable solubility. However, when heated at 70 °C under pressure (due to the use of screw-cap tubes), only the samples at 2.0 and 3.0 mg/mL showed complete solubilization. Considering that a clear solution has the potential to be transferred from batch to continuous-flow reactors, the solutions were checked after 15, 30, and 60 min to assess that the substrate would not precipitate over time. Only the stock at 2.0 mg/mL retained a complete solubility.

The best stoichiometric ratio between the substrate and the acid was evaluated, i.e., 1:1, 1:2, 1:5, and 1:10. The reaction progress was assessed by TLC; reactions with 1:1 and 1:2 showed poor conversion after 24 h, while those with 1:5 and 1:10 showed comparatively better results. For this reason, a 1:5 ratio was selected to perform a model batch reaction under reflux. By monitoring the reaction over time (1, 3, 8, 18, and 24 h), the formation of the aglycone was observed due to the hydrolysis side reaction being triggered by the temperature and residual water in the solvent. After 24 h (40% HPLC molar conversion) (Figure S3.4), the reaction was stopped, and the product was purified (20% isolated yield) and characterized by NMR. Longer reaction times did not evidence an increase in the formation of the desired product.

The same experimental conditions were tested on genistin and glycitin, using octanoic acid as the acyl donor. Reactions with genistin showed the formation of a complex mixture of degradation byproducts due to the high temperature and prolonged reaction time. Glycitin was not reactive under the aforementioned conditions. Notably, the observed results are in accordance with the previous publication on the inhibitory activity of glycitin and genistin on porcine pancreatic lipase (PPL).^43,44^

With the idea of improving the described outcomes of the biocatalyzed acetylation, taking advantage of the advantages associated with continuous biocatalyzed processes, the synthesis of compounds **1**–**6** was transferred to a continuous-flow system. CaLB was packed in a glass column reactor together with powder molecular sieves. A stock solution of the isoflavone (2.0 mg/mL in acetone) was flown through the obtained bioreactor. The residence time and temperature were varied to achieve the highest molar conversion; the results are summarized in Table 2. Once again, the reaction between daidzin and octanoic acid was taken as a model reaction, and the outputs of the reactions were analyzed through HPLC.

**Table 2:** Optimization of Experimental Parameters in Continuous Flow

entry	residence time (min)	temperature (°C)	molar ratio sub./org. acid	molar conversion (%)^a^
1	30	50	1:5	16
2	30	70	1:5	34
3	30	80	1:5	19
4	7	70	1:5	9
5	15	70	1:5	15
6	60	70	1:5	35
7	180	70	1:5	36

a Determined by HPLC.

Keeping the residence time constant at 30 min, the temperature was varied (50, 70, and 80 °C, Table 2, entries 1–3) and 70 °C resulted in the optimal one (Table 2, entry 2). A back-pressure regulator (3 bar) was applied to the system. Then, different residence times were evaluated. As expected, a reduction in the residence time negatively affected the conversion (entries 4 and 5). However, longer residence time did not determine any increase in the conversion, suggesting that an equilibrium is achieved after 30 min (entries 2, 6, 7). A decrease in the ratio between the substrate and the carboxylic acid was also detrimental. Therefore, optimized experimental parameters are reported in entry 2 in Table 2.

Since an excess of organic acid was beneficial to the conversion, an in-line purification procedure using a scavenger ionic resin to remove the unreacted octanoic acid was designed. Different basic resins such as Amberlite IRA-67 free base, Amberlyst A21 free base, Ambersep 900 hydroxide form, Amberlite IRA-400(Cl) ion-exchange resin, and QuadraPure BZA were evaluated to identify the best one able to catch the acid leaving in solution, the product, or the substrate. Therefore, 20.0 mg of resin was placed in a 4 mL vial together with 2.0 mg/mL solution of daidzin in acetone (2.0 mL). The system was left under gentle stirring at room temperature, and the outcome was assessed through TLC after 30 and 60 min. Since only Amberlite IRA-400(Cl) ion-exchange resin and QuadraPure BZA did not react with the glycoside, these were subsequently tested for their ability to catch the organic acid. QuadraPure BZA was unable to catch octanoic acid, while Amberlite IRA-400(Cl) ion-exchange resin successfully removed it from the solution.

**Scheme 1:**
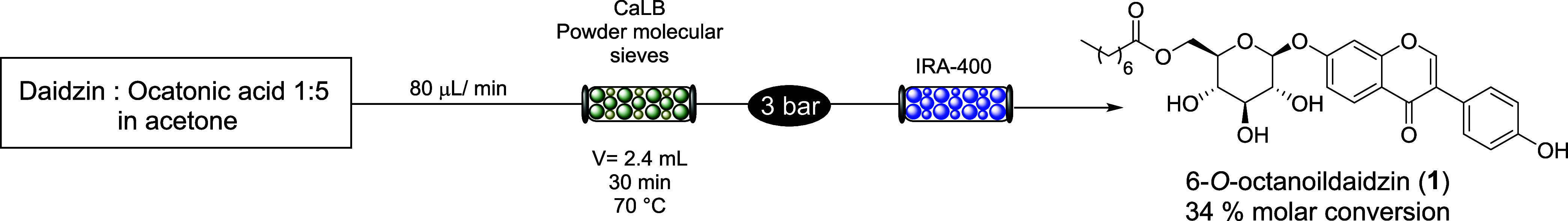
Schematic Representation of the Flow Synthesis of Compound **1**

The final continuous-flow setup for the synthesis of 6-*O*-octanoildaidzin (**1**) is shown in Scheme 1. It is worth noting that different from the batch reaction, no aglycone was found in the output, probably due to the shorter reaction and heating times (30 min versus 18 h), facilitating the purification procedure.

Using the same reaction conditions, the continuous synthesis of compounds **2** and **3** has been performed, leading to 37% m.c. for compound **2** (Figure S3.12) and 14% m.c. for compound **3** (Figure S3.13). Comparing the results with the batch procedure for compound **1**, a slightly better yield was obtained after purification using the continuous-flow protocol (24 vs 20%); however, it must be observed that this result is obtained after a drastic reduction in reaction time (30 min vs 18 h) with a consequent 9-fold increase in specific reaction rates (0.15 vs 0.017 μmol/(min·g)). Again, no aglycone was found in the existing solution.

Afterward, the optimized flow protocol has been also attempted for the synthesis of compounds **4**–**6**, providing lower molar conversion compared to daidzin: 16% for **4** (Figure S3.14), 11% for **5** (Figure S3.15), and 8% for **6** (Figure S3.16). Unfortunately, NMR analysis showed a mixture of the desired product together with structural isomers, possibly due to acyl chain migration from *O*-6 to *O*-4 positions of glucose (the phenomenon was also observed in the absence of IRA-400) (Figures S4.13–S4.15).^45^

## 4 Conclusions

Soybean residues and waste are a valuable source of bioactive compounds that could be further valorized as high-value-added compounds. In particular, glycoside isoflavones such as daidzin, genistin, and glycitin are known for their antioxidant power; however, the exploitation of their potential is often hindered by their physical and chemical properties (i.e., high hydrophilicity, modest chemical stability). In this work, two different biocatalytic approaches were developed to increase their lipophilicity. First, HOR has been produced and used to obtain the aglycones (daidzein, genistein, and glycitein) with full conversion and from good to high yields. Successively, commercially available CaLB has been selected as the suitable immobilized enzyme to perform the lipophilization of the primary alcohol on C6 of glucose with different organic acids (butanoic, hexanoic, and octanoic acids). After optimization of the batch conditions on the model reaction between daidzin and octanoic acid, the same experimental parameters have been used for genistin and glycitin. Unfortunately, a complex mixture has been obtained with the first one, whereas no reactivity has been observed for the latter. These results are in accordance with a previous publication on the inhibitory activity of glycitin and genistin on porcine pancreatic lipase (PPL). The formation of aglycones as side products has always been observed. With the aim of increasing the productivity, reducing the manual handling, and improving the automation of the process, the reaction was submitted to a flow shift. The temperature, residence time, and stoichiometric ratio have been evaluated and optimized (30 min, 70 °C, 1:5 molar ratio between isoflavone and organic acid). Compared to the batch procedure, compound **1** was obtained after a drastic reduction in reaction time (30 min vs 18 h) with a consequent 9-fold increase in specific reaction rates (0.15 vs 0.017 μmol/(min·g)) and no aglycone was formed. The same process was applied successfully to the synthesis of compounds **2** and **3**. Moreover, an in-line purification step has been added downstream the process using Amberlite IRA-400(Cl) ion-exchange resin to trap the exceeding organic acid, further increasing the system automation.

## Supporting Information Available

SDS-PAGE of purified HOR (Figure S1); calculated molecular weights, clog *P*, and clog *S* (Table S1); HPLC chromatograms (Figures S3.1–S3.16); and ^1^H NMR and ^13^C NMR spectra (Figures S4.1–S4.15) (PDF)

## References

[ref1] Medic J.; Atkinson C.; Hurburgh C. R. Current Knowledge in Soybean Composition. J. Am. Oil Chem. Soc. 2014, 91 (3), 363–384. 10.1007/s11746-013-2407-9.

[ref2] Anderson E. J.; Ali M. L.; Beavis W. D.; Chen P.; Clemente T. E.; Diers B. W.; Graef G. L.; Grassini P.; Hyten D. L.; McHale L. K.; Nelson R. L.; Parrott W. A.; Patil G. B.; Stupar R. M.; Tilmon K. J. Soybean [*Glycine max* (L.) Merr.] Breeding: History, Improvement, Production and Future Opportunities. In Advances in Plant Breeding Strategies: Legumes; Al-Khayri J.; Jain S.; Johnson D., Eds.; Springer: Cham, 2019; pp 431–516.

[ref3] Nile S. H.; Venkidasamy B.; Samynathan R.; Nile A.; Shao K.; Chen T.; Sun M.; Khan M. U.; Dutta N.; Thiruvengadam M.; Shariati M. A.; Rebezov M.; Kai G. Soybean Processing Wastes: Novel Insights on Their Production, Extraction of Isoflavones, and Their Therapeutic Properties. J. Agric. Food Chem. 2022, 70 (23), 6849–6863. 10.1021/acs.jafc.1c04927. 34645264

[ref4] Seo W. D.; Kang J. E.; Choi S. W.; Lee K. S.; Lee M. J.; Park K. Do.; Lee J. H. Comparison of Nutritional Components (Isoflavone, Protein, Oil, and Fatty Acid) and Antioxidant Properties at the Growth Stage of Different Parts of Soybean [Glycine Max (L.) Merrill]. Food Sci. Biotechnol. 2017, 26 (2), 339–347. 10.1007/s10068-017-0046-x. 30263548 PMC6049444

[ref5] Sirtori C. R. Risks and Benefits of Soy Phytoestrogens in Cardiovascular Diseases, Cancer, Climacteric Symptoms and Osteoporosis. Drug Saf. 2001, 24 (9), 665–682. 10.2165/00002018-200124090-00003. 11522120

[ref6] Kim I.-S. Current Perspectives on the Beneficial Effects of Soybean Isoflavones and Their Metabolites for Humans. Antioxidants 2021, 10, 1064 10.3390/antiox10071064. 34209224 PMC8301030

[ref7] Jucá M. M.; Cysne Filho F. M. S.; de Almeida J. C.; da Silva Mesquita D.; de Moraes Barriga J. R.; Dias K. C. F.; Barbosa T. M.; Vasconcelos L. C.; Leal L. K. A. M.; Ribeiro J. E.; Vasconcelos S. M. M. Flavonoids: biological activities and therapeutic potential. Nat. Prod. Res. 2020, 34, 692–705. 10.1080/14786419.2018.1493588. 30445839

[ref8] Li C.; Dai T.; Chen J.; Chen M.; Liang R.; Liu C.; Du L.; McClements D. J. Modification of Flavonoids: Methods and Influences on Biological Activities. Crit. Rev. Food Sci. Nutr. 2023, 63 (31), 10637–10658. 10.1080/10408398.2022.2083572. 35687361

[ref9] Contente M. L.; Annunziata F.; Cannazza P.; Donzella S.; Pinna C.; Romano D.; Tamborini L.; Barbosa F. G.; Molinari F.; Pinto A. Biocatalytic Approaches for an Efficient and Sustainable Preparation of Polyphenols and Their Derivatives. J. Agric. Food Chem. 2021, 69 (46), 13669–13681. 10.1021/acs.jafc.1c05088. 34762407

[ref10] Mardani M.; Badakné K.; Farmani J.; Shahidi F. Enzymatic Lipophilization of Bioactive Compounds with High Antioxidant Activity: A Review. Crit. Rev. Food Sci. Nutr. 2024, 64 (15), 4977–4994. 10.1080/10408398.2022.2147268. 36419380

[ref11] Vitale D. C.; Piazza C.; Melilli B.; Drago F.; Salomone S. Isoflavones: Estrogenic Activity, Biological Effect and Bioavailability. Eur. J. Drug Metab. Pharmacokinet. 2013, 38 (1), 15–25. 10.1007/s13318-012-0112-y. 23161396

[ref12] Canaan J. M. M.; Brasil G. S. A. P.; de Barros N. R.; Mussagy C. U.; Guerra N. B.; Herculano R. D. Soybean Processing Wastes and Their Potential in the Generation of High Value Added Products. Food Chem. 2022, 373, 131476 10.1016/j.foodchem.2021.131476. 34731815

[ref13] Wang H. J.; Murphy P. A. Isoflavone Content in Commercial Soybean Foods. J. Agric. Food Chem. 1994, 42 (8), 1666–1673. 10.1021/jf00044a016.

[ref14] Bryan M. C.; Dillon B.; Hamann L. G.; Hughes G. J.; Kopach M. E.; Peterson E. A.; Pourashraf M.; Raheem I.; Richardson P.; Richter D.; Sneddon H. F. Sustainable Practices in Medicinal Chemistry: Current State and Future Directions. J. Med. Chem. 2013, 56 (15), 6007–6021. 10.1021/jm400250p. 23586692

[ref15] Rozzell J. D. Biocatalysis at Commercial Scale: Myths and Realities. Chim. Oggi 1999, 17 (5–6), 42–47.

[ref16] Sheldon R. A.; Woodley J. M. Role of Biocatalysis in Sustainable Chemistry. Chem. Rev. 2018, 118 (2), 801–838. 10.1021/acs.chemrev.7b00203. 28876904

[ref17] Sheldon R. A.; Brady D. Broadening the Scope of Biocatalysis in Sustainable Organic Synthesis. ChemSusChem 2019, 12 (13), 2859–2881. 10.1002/cssc.201900351. 30938093

[ref18] Tamborini L.; Fernandes P.; Paradisi F.; Molinari F. Flow Bioreactors as Complementary Tools for Biocatalytic Process Intensification. Trends Biotechnol. 2018, 36 (1), 73–88. 10.1016/j.tibtech.2017.09.005. 29054312

[ref19] Benítez-Mateos A. I.; Contente M. L.; Roura Padrosa D.; Paradisi F. Flow Biocatalysis 101: Design, Development and Applications. React. Chem. Eng. 2021, 6 (4), 599–611. 10.1039/D0RE00483A.

[ref20] De Santis P.; Meyer L. E.; Kara S. The Rise of Continuous Flow Biocatalysis-Fundamentals, Very Recent Developments and Future Perspectives. React. Chem. Eng. 2020, 5 (12), 2155–2184. 10.1039/D0RE00335B.

[ref21] Crotti M.; Robescu M. S.; Bolivar J. M.; Ubiali D.; Wilson L.; Contente M. L. What’s New in Flow Biocatalysis? A Snapshot of 2020–2022. Front. Catal. 2023, 3, 1154452 10.3389/fctls.2023.1154452.

[ref22] Walle T. Methylation of Dietary Flavones Greatly Improves Their Hepatic Metabolic Stability and Intestinal Absorption. Mol. Pharmaceutics 2007, 4 (6), 826–832. 10.1021/mp700071d. 17958394

[ref23] Bayrasy C.; Chabi B.; Laguerre M.; Lecomte J.; Jublanc É.; Villeneuve P.; Wrutniak-Cabello C.; Cabello G. Boosting Antioxidants by Lipophilization: A Strategy to Increase Cell Uptake and Target Mitochondria. Pharm. Res. 2013, 30 (8), 1979–1989. 10.1007/s11095-013-1041-4. 23604925

[ref24] Jasińska K.; Fabiszewska A.; Białecka-Florjańczyk E.; Zieniuk B. Mini-Review on the Enzymatic Lipophilization of Phenolics Present in Plant Extracts with the Special Emphasis on Anthocyanins. Antioxidants 2022, 11 (8), 1528 10.3390/antiox11081528. 36009246 PMC9405086

[ref25] Miltyk W.; Craciunescu C. N.; Fischer L.; Jeffcoat R. A.; Koch M. A.; Lopaczynski W.; Mahoney C.; Crowell J.; Paglieri J.; Zeisel S. H. Lack of significant genotoxicity of purified soy isoflavones (genistein, daidzein, and glycitein) in 20 patients with prostate cancer. Am. J. Clin. Nutr. 2003, 77 (4), 875–882. 10.1093/ajcn/77.4.875. 12663286

[ref26] Bloedon L. T.; Jeffcoat R.; Lopaczynski W.; Schell M. J.; Black T. M.; Dix K. J.; Thomas B. F.; Albright C.; Busby M. G.; Crowell J. A.; Zeisel S. H. Safety and pharmacokinetics of purified soy isoflavones: single-dose administration to postmenopausal women. Am. J. Clin. Nutr. 2002, 76 (5), 1126–1137. 10.1093/ajcn/76.5.1126. 12399289

[ref27] Kornpointner C.; Scheibelreiter J.; Halbwirth H. Snailase: A Promising Tool for the Enzymatic Hydrolysis of Flavonoid Glycosides From Plant Extracts. Front. Plant Sci. 2022, 13 (13), 889184 10.3389/fpls.2022.889184. 35755698 PMC9218754

[ref28] Marathe S. J.; Dedhia N.; Singhal R. S. Esterification of sugars and polyphenols with fatty acids: techniques, bioactivities, and applications. Curr. Opin. Food Sci. 2022, 43, 163–173. 10.1016/j.cofs.2021.12.008.

[ref29] Annunziata F.; Contente M. L.; Anzi V.; Donzella D.; Conti P.; Molinari F.; Martino P. A.; Meroni G.; Sora V. M.; Tamborini L.; Pinto A. Enzymatic continuous-flow preparation of nature-inspired phenolic esters as antiradical and antimicrobial agents. Food Chem. 2022, 390, 133195 10.1016/j.foodchem.2022.133195. 35594770

[ref30] Meng Q. H.; Lewis P.; Wähälä K.; Adlercreutz H.; Tikkanen M. J. Incorporation of esterified soybean isoflavones with antioxidant activity into low density lipoprotein. Biochim. Biophys. Acta, Mol. Cell Biol. Lipids 1999, 1438 (3), 369–376. 10.1016/S1388-1981(99)00062-1. 10366779

[ref31] Uyama H.; Kobayashi S. Enzyme-catalyzed polymerization to functional polymers. J. Mol. Catal. B: Enzym. 2002, 19–20, 117–127. 10.1016/S1381-1177(02)00158-3.

[ref32] Delgado L.; Heckmann C. M.; Di Pisa F.; Gourlay L.; Paradisi F. Release of Soybean Isoflavones by Using a β-Glucosidase from Alicyclobacillus Herbarius. ChemBioChem 2021, 22 (7), 1223–1231. 10.1002/cbic.202000688. 33237595 PMC8048572

[ref33] Delgado L.; Parker M.; Fisk I.; Paradisi F. Performance of the Extremophilic Enzyme BglA in the Hydrolysis of Two Aroma Glucosides in a Range of Model and Real Wines and Juices. Food Chem. 2020, 323, 126825 10.1016/j.foodchem.2020.126825. 32335459

[ref34] Ketudat Cairns J. R.; Esen A. β-Glucosidases. Cell. Mol. Life Sci. 2010, 67 (20), 3389–3405. 10.1007/s00018-010-0399-2. 20490603 PMC11115901

[ref35] Suzuki H.; Takahashi S.; Watanabe R.; Fukushima Y.; Fujita N.; Noguchi A.; Yokoyama R.; Nishitani K.; Nishino T.; Nakayama T. An Isoflavone Conjugate-Hydrolyzing β-Glucosidase from the Roots of Soybean (Glycine Max) Seedlings: Purification, Gene Cloning, Phylogenetics, and Cellular Localization. J. Biol. Chem. 2006, 281 (40), 30251–30259. 10.1074/jbc.M605726200. 16891302

[ref36] Chuankhayan P.; Hua Y.; Svasti J.; Sakdarat S.; Sullivan P. A.; Ketudat Cairns J. R. Purification of an Isoflavonoid 7-O-β-Apiosyl-Glucoside β-Glycosidase and Its Substrates from Dalbergia Nigrescens Kurz. Phytochemistry 2005, 66 (16), 1880–1889. 10.1016/j.phytochem.2005.06.024. 16098548

[ref37] Chuankhayan P.; Rimlumduan T.; Svasti J.; Ketudat Cairns J. R. Hydrolysis of Soybean Isoflavonoid Glycosides by Dalbergia β-Glucosidases. J. Agric. Food Chem. 2007, 55 (6), 2407–2412. 10.1021/jf062885p. 17311399

[ref38] Chuankhayan P.; Rimlumduan T.; Tantanuch W.; Mothong N.; Kongsaeree P. T.; Metheenukul P.; Svasti J.; Jensen O. N.; Cairns J. R. K. Functional and Structural Differences between Isoflavonoid β-Glycosidases from Dalbergia Sp. Arch. Biochem. Biophys. 2007, 468 (2), 205–216. 10.1016/j.abb.2007.09.015. 17998137

[ref39] Ismail B.; Hayes K. β-Glycosidase Activity toward Different Glycosidic Forms of Isoflavones. J. Agric. Food Chem. 2005, 53 (12), 4918–4924. 10.1021/jf0404694. 15941336

[ref40] Colacicco A.; Catinella G.; Pinna C.; Pellis A.; Farris S.; Tamborini L.; Dallavalle S.; Molinari F.; Contente M. L.; Pinto A. Flow Bioprocessing of Citrus Glycosides for High-Value Aglycone Preparation. Catal. Sci. Technol. 2023, 13 (15), 4348–4352. 10.1039/D3CY00603D.

[ref41] Byrne F. P.; Clark J. H.; Angelici C.; de Jong E.; Farmer T. J. Greenness Assessment and Synthesis for the Bio-Based Production of the Solvent 2,2,5,5-Tetramethyloxolane (TMO). Sustainable Chem. 2021, 2 (3), 392–406. 10.3390/suschem2030023.

[ref42] Casas-Godoy L.; Duquesne S.; Bordes F.; Sandoval G.; Marty A. Lipases: An Overview. In Lipases and Phospholipases. Methods in Molecular Biology; Sandoval G., Ed.; Humana Press: Totowa, 2012; Vol. 861, pp 3–30. 10.1007/978-1-61779-600-5_1 22426709

[ref43] Kenneth D. S. Phytoestrogens: The Biochemistry, Physiology, and Implications for Human Health of Soy Isoflavones. Am. J. Clin. Nutr. 1998, 68, 1333S–1346S. 10.1093/ajcn/68.6.1333S. 9848496

[ref44] Li M. M.; Chen Y. T.; Ruan J. C.; Wang W. J.; Chen J. G.; Zhang Q. F. Structure-Activity Relationship of Dietary Flavonoids on Pancreatic Lipase. Curr. Res. Food Sci. 2023, 6, 100424 10.1016/j.crfs.2022.100424. 36618100 PMC9813676

[ref45] Yerramsetty V.; Mathias K.; Bunzel M.; Ismail B. Detection and Structural Characterization of Thermally Generated Isoflavone Malonylglucoside Derivatives. J. Agric. Food Chem. 2011, 59 (1), 174–183. 10.1021/jf103564y. 21141961

