# Supplementary material for: Enzymatic Deglycosylation and Lipophilization of Soy Glycosides into Value-Added Compounds for Food and Cosmetic Applications
Source: ACS Omega. 2025 Mar 20;10(12):12417–24. doi: 10.1021/acsomega.4c11325 (PMC11966307; doi:10.1021/acsomega.4c11325)
Supplement: Supplementary file 1 — ao4c11325_si_001.pdf [file ao4c11325_si_001.pdf]

Enzymatic deglycosylation and lipophilization of soy glycosides into value-added compounds for food and cosmetic applications

Matteo Corti,[a†] Francesca Annunziata,[a†] Agostina Colacicco,[a] Lucia Tamborini,[b] Francesco Molinari,[a] Martina Letizia Contente,[a*] Andrea Pinto [a]

[†] These authors contributed equally to this work

[a] Department of Food Environmental and Nutritional Sciences (DeFENS), University of Milan, via Celoria 2, 20133 Milano

[b] Department of Pharmaceutical Sciences (DISFARM), University of Milan, via Mangiagalli 25, 20133 Milano

Correspondence: martina.contente@unimi.it

1. Protein purification

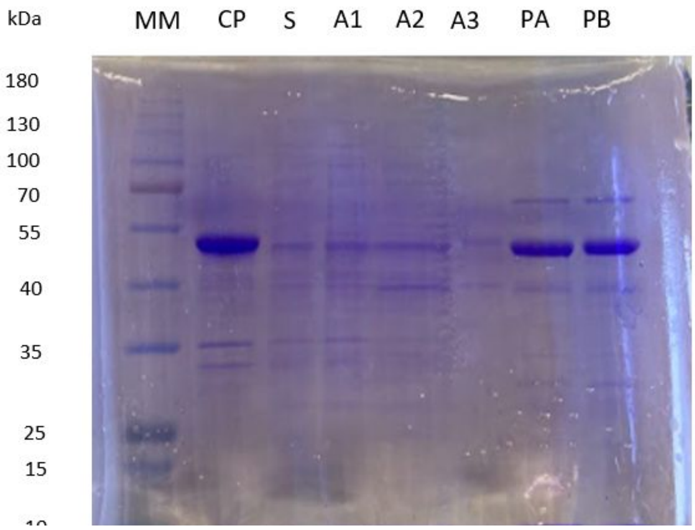

Figure S1: SDS-PAGE of the purified HOR: MM = molecular markers; CP = cellular pellet debris; S = surnatant; A1 = aspecific 1; A2 = aspecific 2; A3 = aspecific 3; PA,PB = HOR purified.

2. Properties of the products

Compound	Molecular Weight (Da)	clogS	clogP
**Daidzin**	416.381	-2.909	-0.0164
**Daidzein**	254.240	-3.023	1.9729
**6-O-butanoildaidzin**	486.471	-3.859	1.3770
**6-O-hexanoildaidzin**	514.525	-4.399	2.2858
**6-O-octanoildaidzin**	542.579	-4.939	3.1946
**Genistin**	430.408	-2.616	0.2190
**Genistein**	268.267	-2.730	2.2083
**6-O-butanoilgenistin**	500.498	-3.566	1.6124
**6-O-hexanoilgenistin**	528.552	-4.106	2.5212
**6-O-octanoilgenistin**	556.606	-4.646	3.4300

Table S1: clogS and clogP of the glycosides, aglycones and products of the lipophilization reactions.

3. HPLC chromatograms

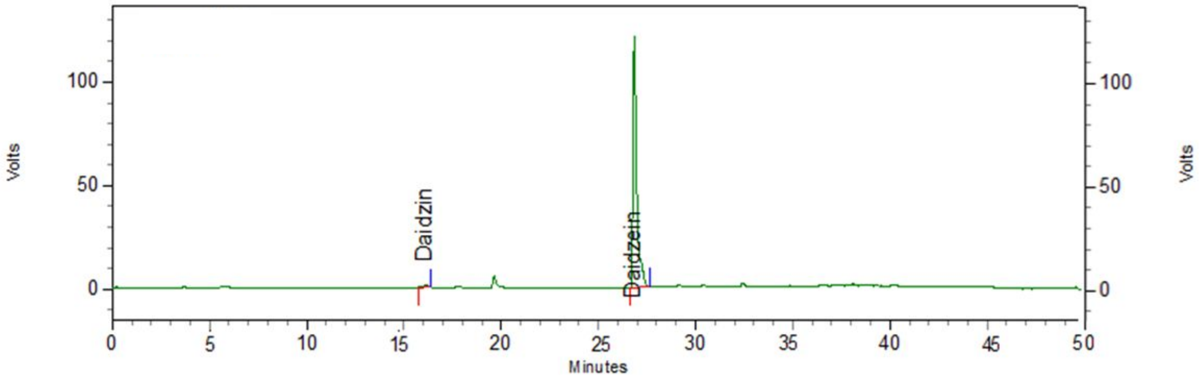

Figure S3.1: Chromatogram of the hydrolysis of daidzin after 60 min (batch).

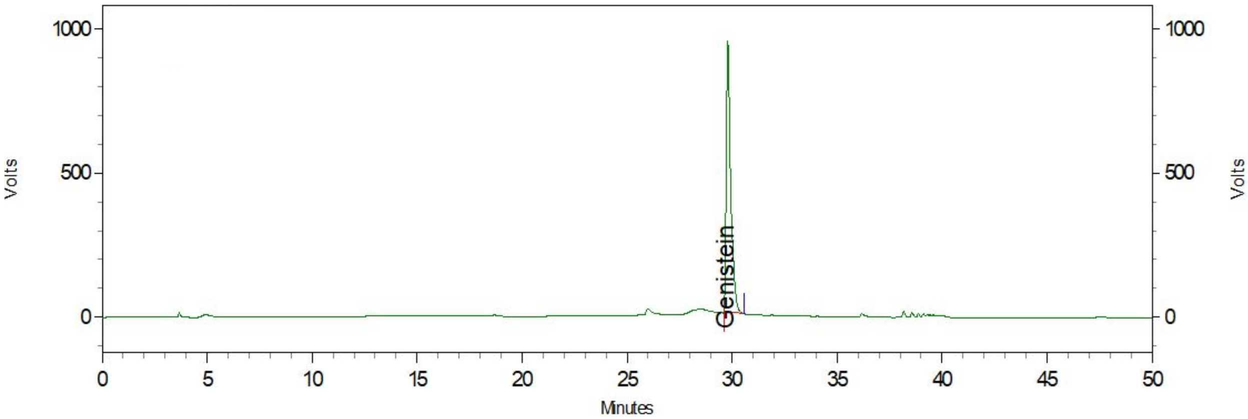

Figure S3.2: Chromatogram of the hydrolysis of genistin after 15 min (batch).

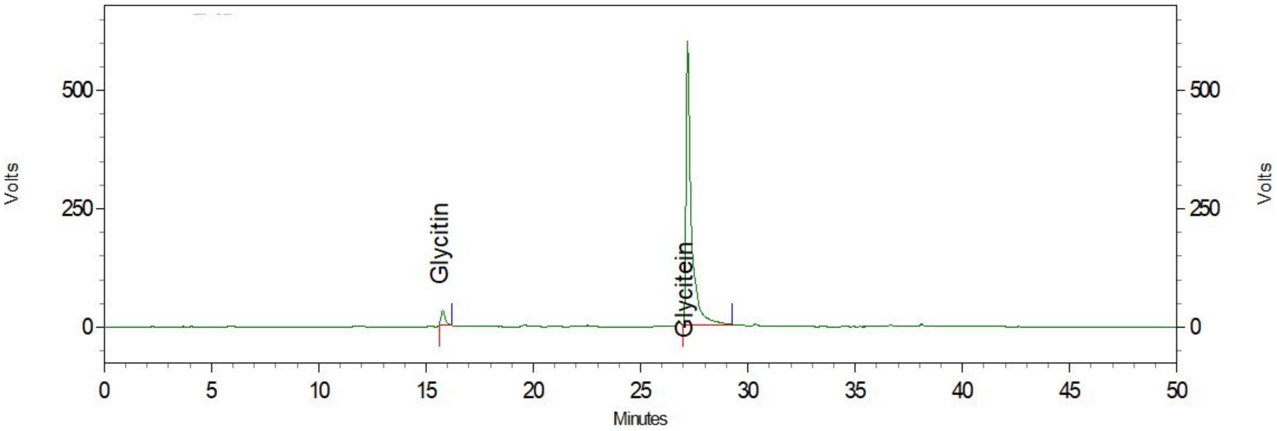

Figure S3.1: Chromatogram of the hydrolysis of glycitin after 180 min (batch).

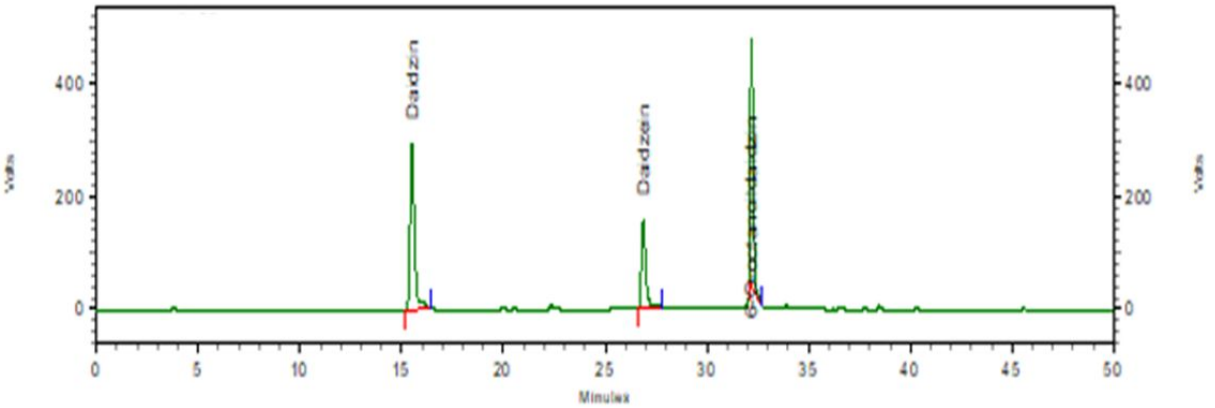

Figure S3.4: Chromatogram of the lipophilization of daidzin with octanoic acid in batch after 18 h (batch).

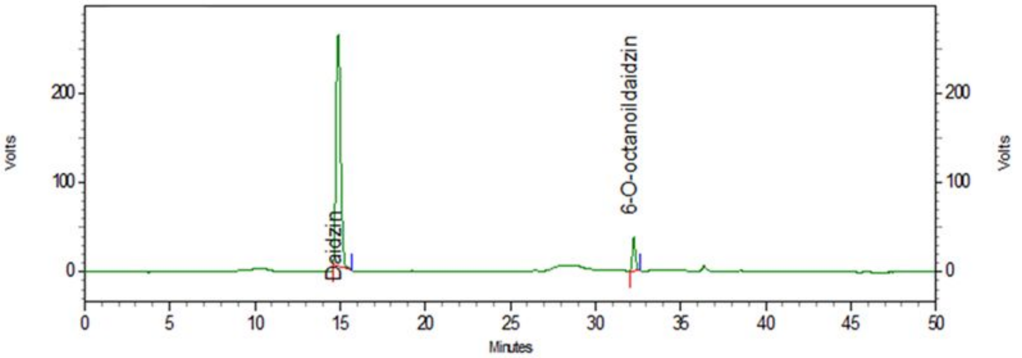

Figure S3.5: Chromatogram of the lipophilization of daidzin with octanoic acid in flow stream, with a residence time of 7 min at 70 °C.

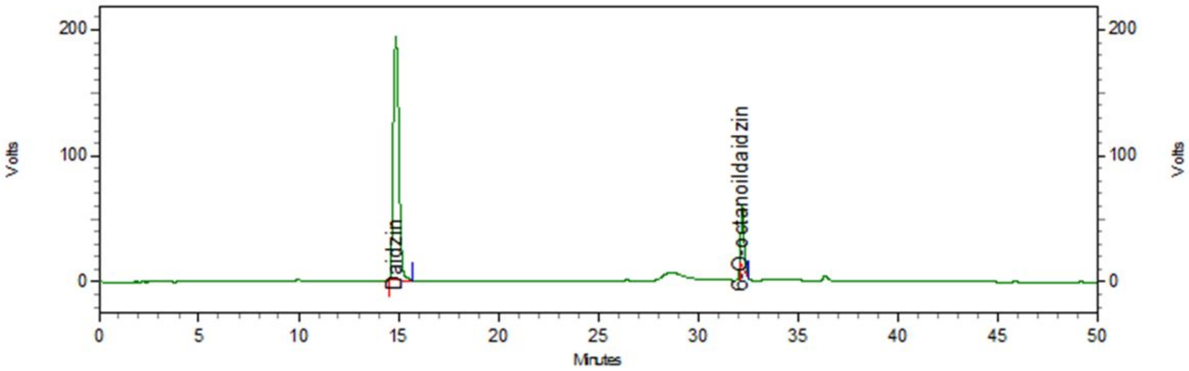

Figure S3.6: Chromatogram of the lipophilization of daidzin with octanoic acid in flow stream, with a residence time of 15 min at 70 °C.

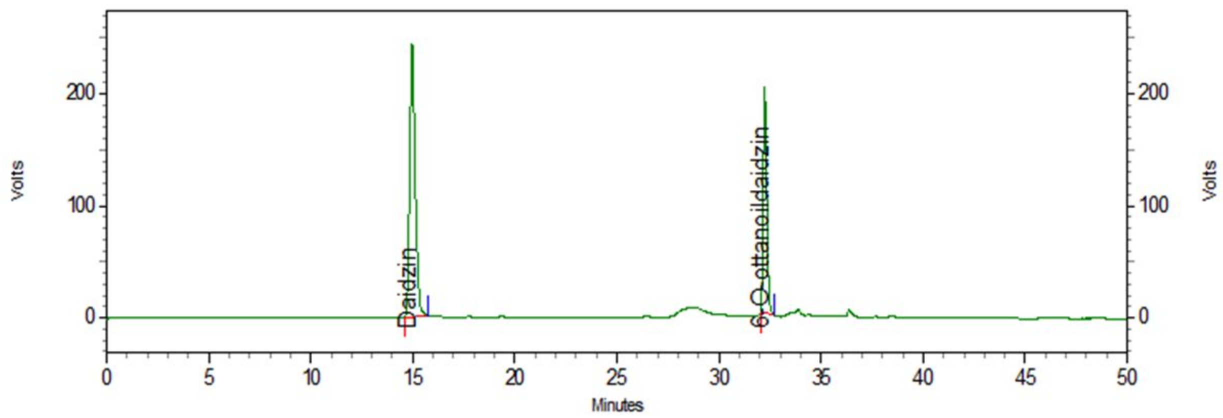

Figure S3.7: Chromatogram of the lipophilization of daidzin with octanoic acid in flow stream, with a residence time of 30 min at 70 °C.

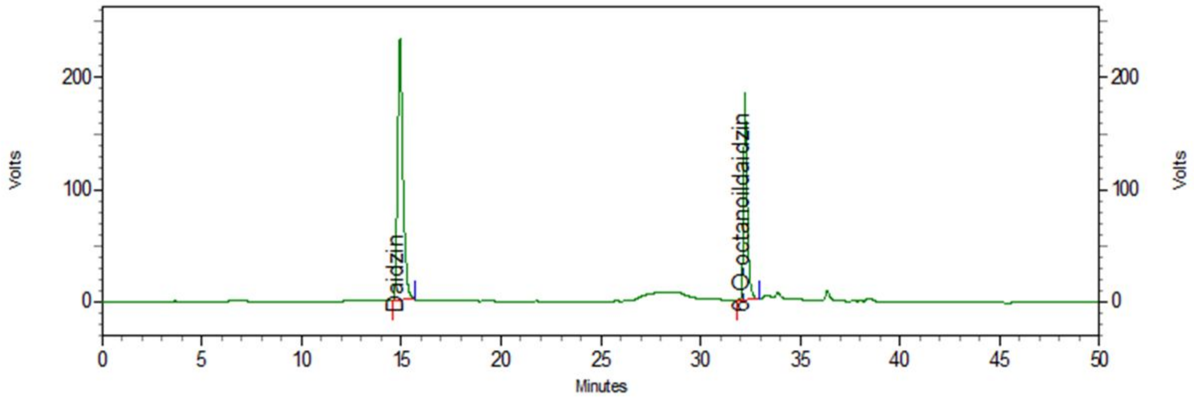

Figure S3.8: Chromatogram of the lipophilization of daidzin with octanoic acid in flow stream, with a residence time of 60 min at 70 °C.

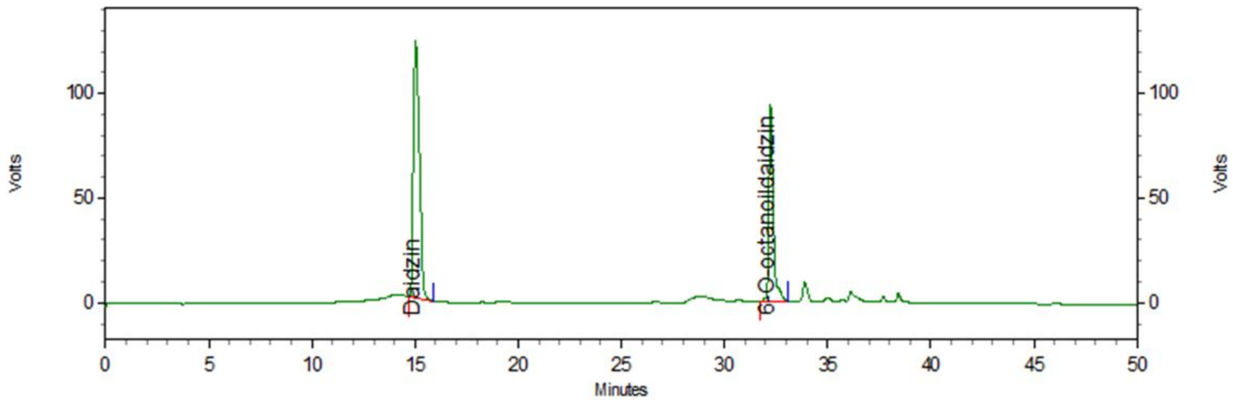

Figure S3.9: Chromatogram of the lipophilization of daidzin with octanoic acid in flow stream, with a residence time of 180 min at 70 °C.

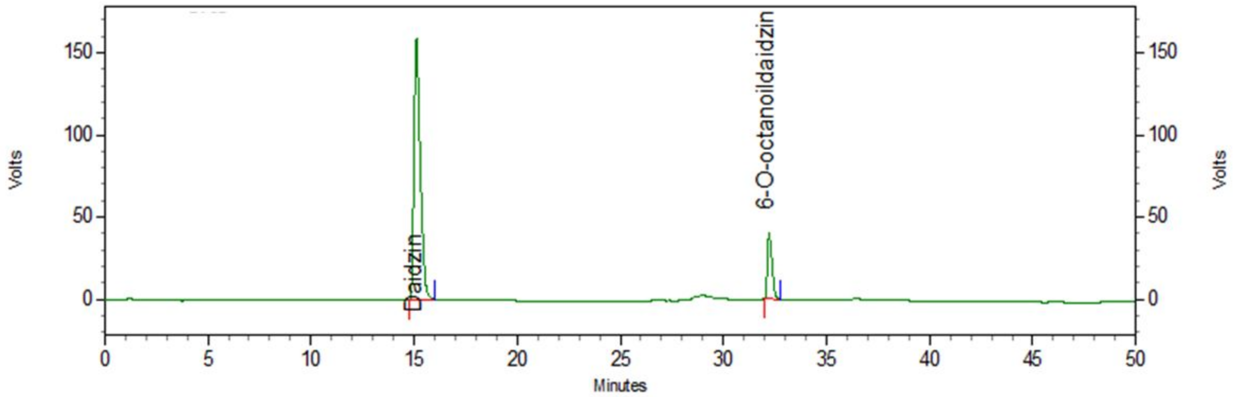

Figure S3.10: Chromatogram of the lipophilization of daidzin with octanoic acid in flow stream with a residence time of 30 min at 50 °C.

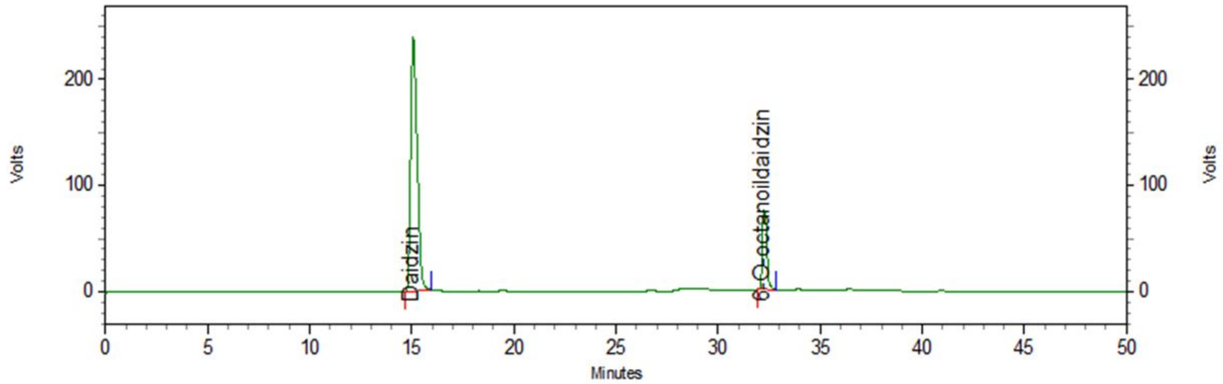

Figure S3.11: Chromatogram of the lipophilization of daidzin with octanoic acid in flow stream with a residence time of 30 min at 80 °C.

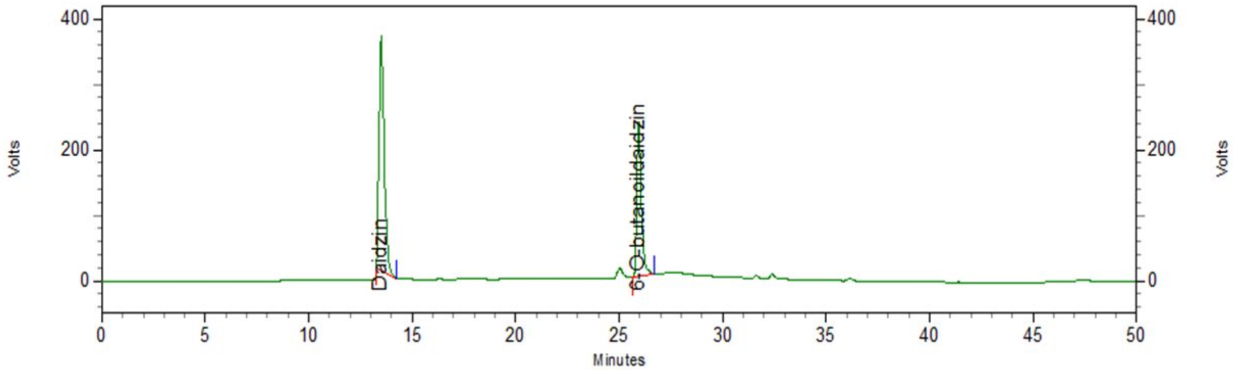

Figure S3.12: Chromatogram of the lipophilization of daidzin with butanoic acid in flow stream with a residence time of 30 min at 70 °C.

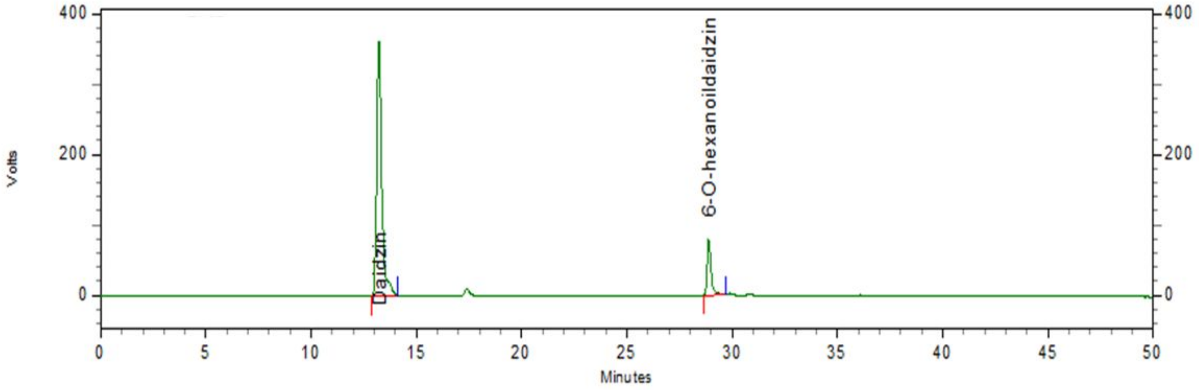

Figure S3.13: Chromatogram of the lipophilization of daidzin with hexanoic acid in flow stream with a residence time of 30 min at 70 °C.

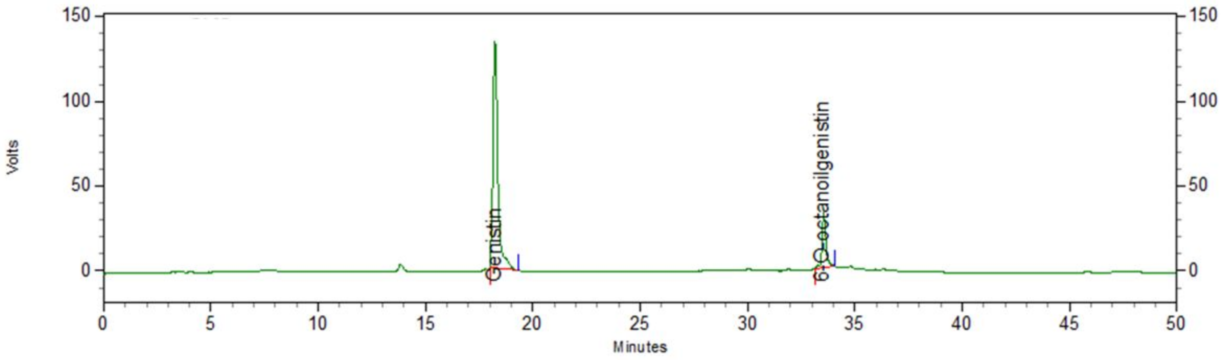

Figure S3.14: Chromatogram of the lipophilization of genistin with octanoic acid in flow stream with a residence time of 30 min at 70 °C.

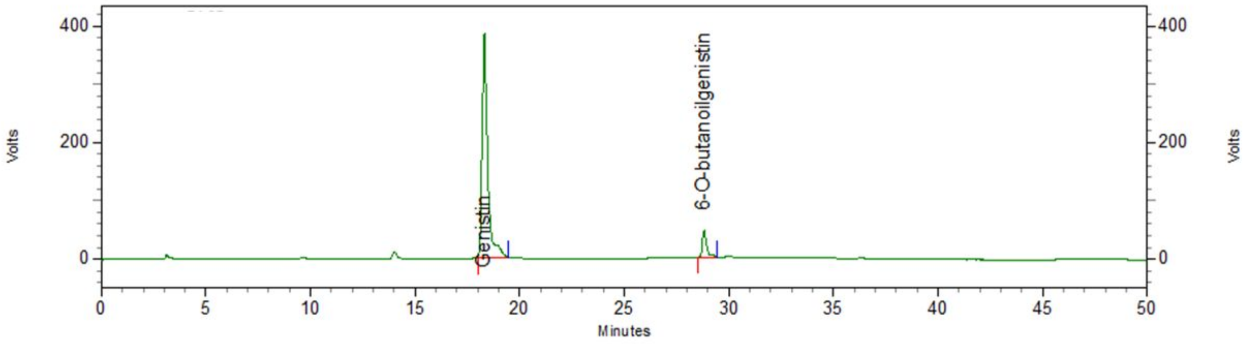

Figure S3.15: Chromatogram of the lipophilization of genistin with butanoic acid in flow stream with a residence time of 30 min at 70 °C

.

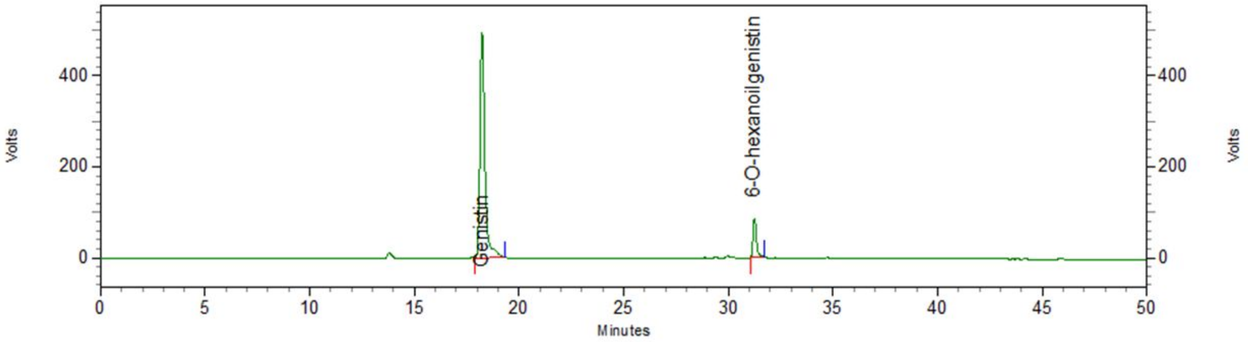

Figure S3.16: Chromatogram of the lipophilization of genistin with hexanoic acid in flow stream with a residence time of 30 min at 70 °C.

4. NMR spectra

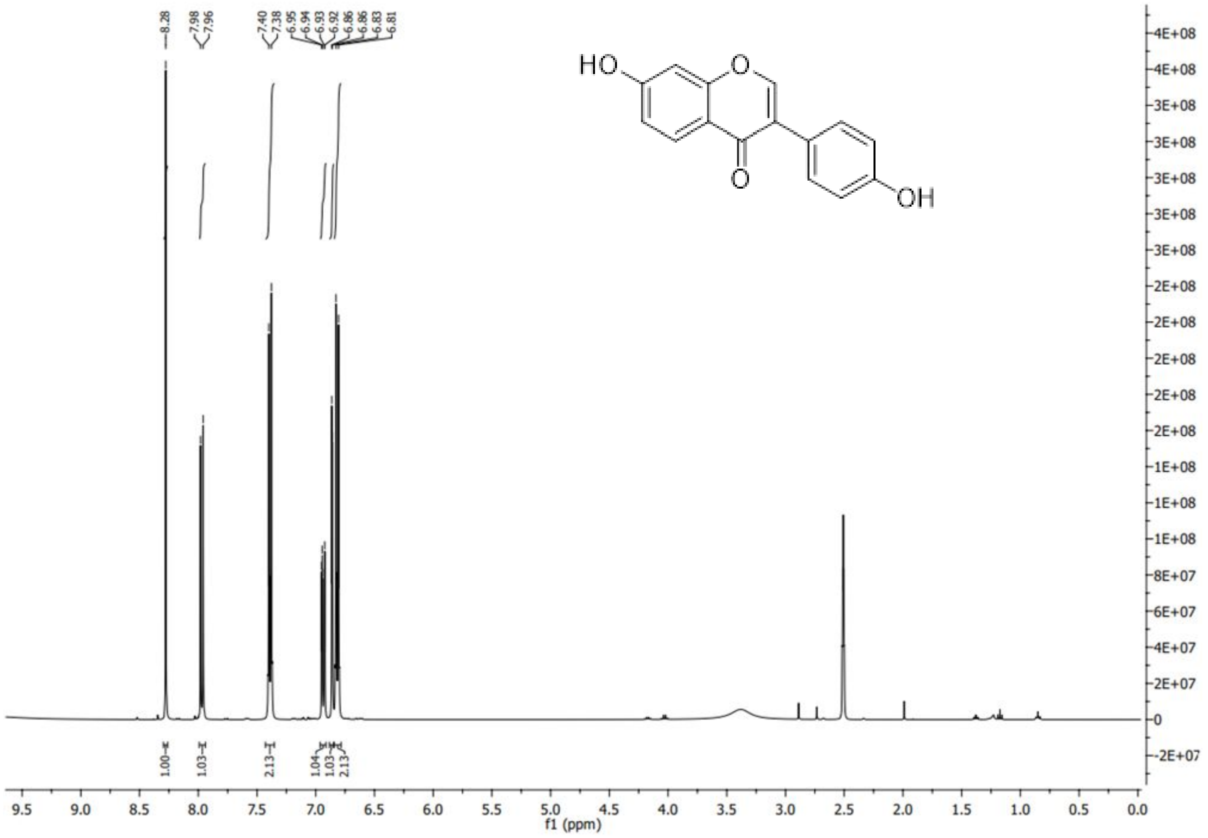

Figure S4.1: 1H-NMR spectrum of daidzein.

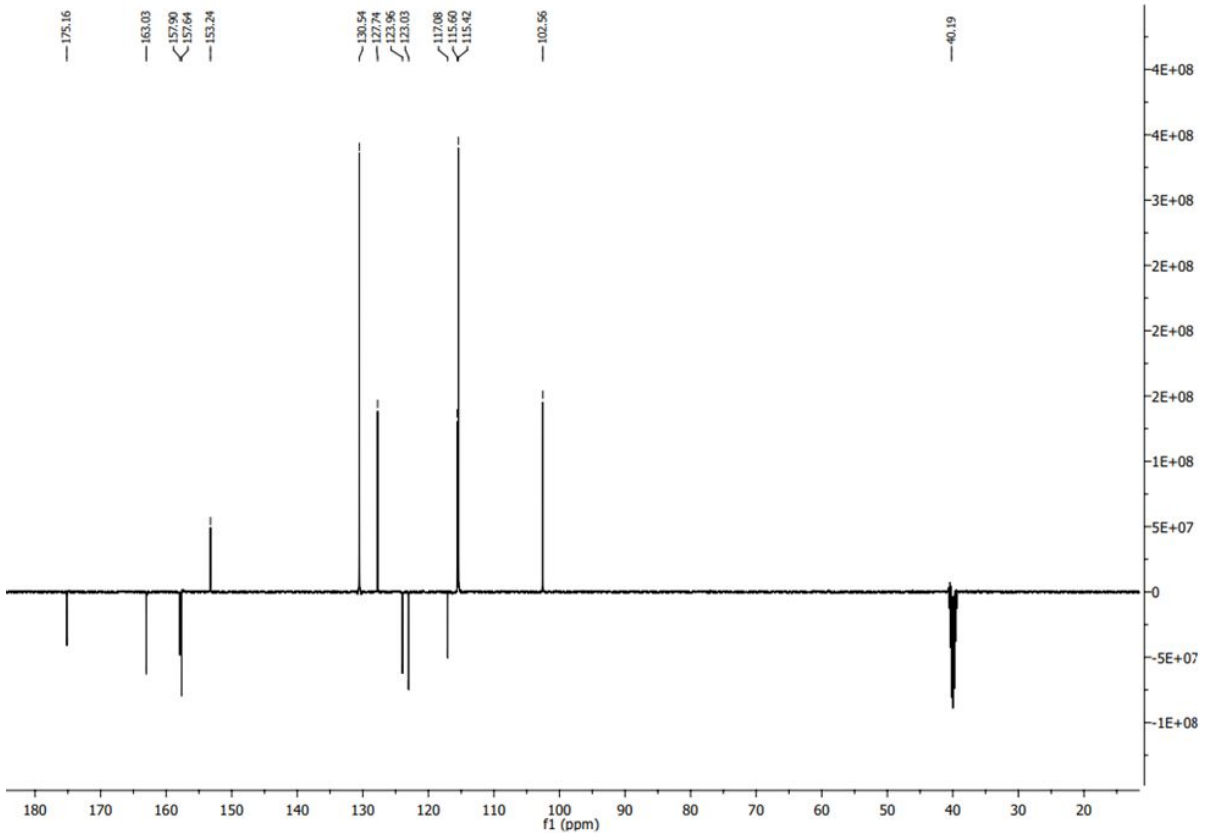

Figure S4.2: 13C-NMR spectrum of daidzein

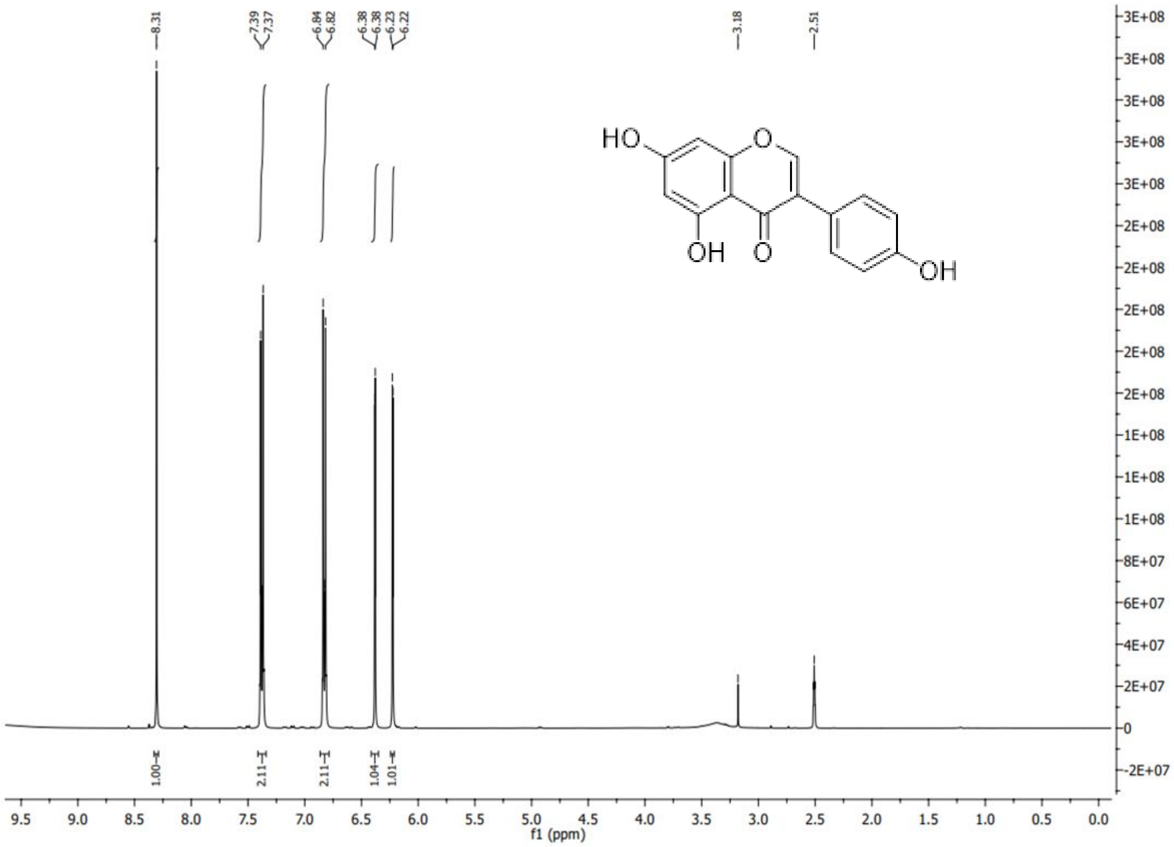

FigureS4.3: ^{1}H-NMR spectrum of genistein.

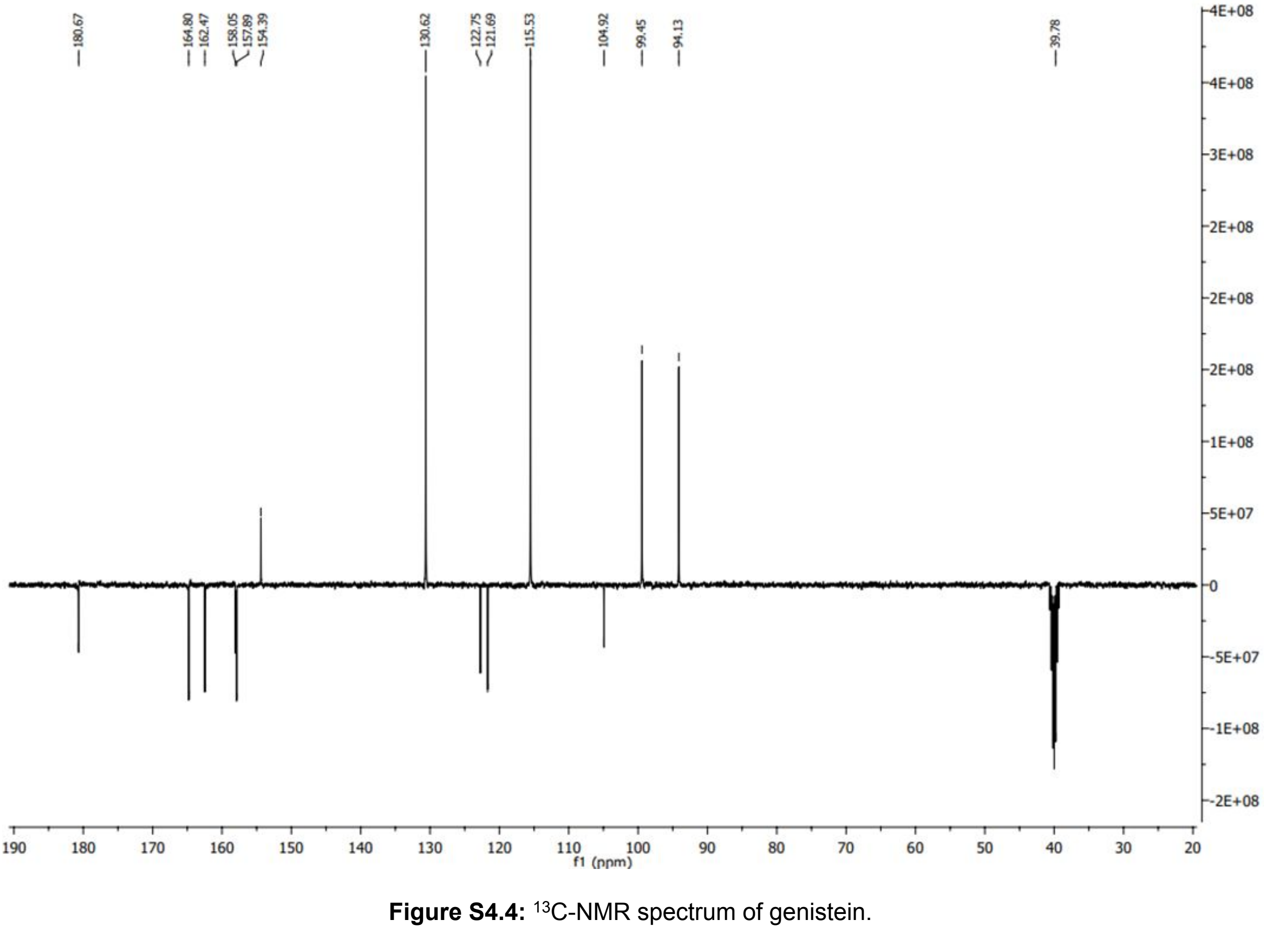

Figure S4.4: ^{13}C-NMR spectrum of genistein.

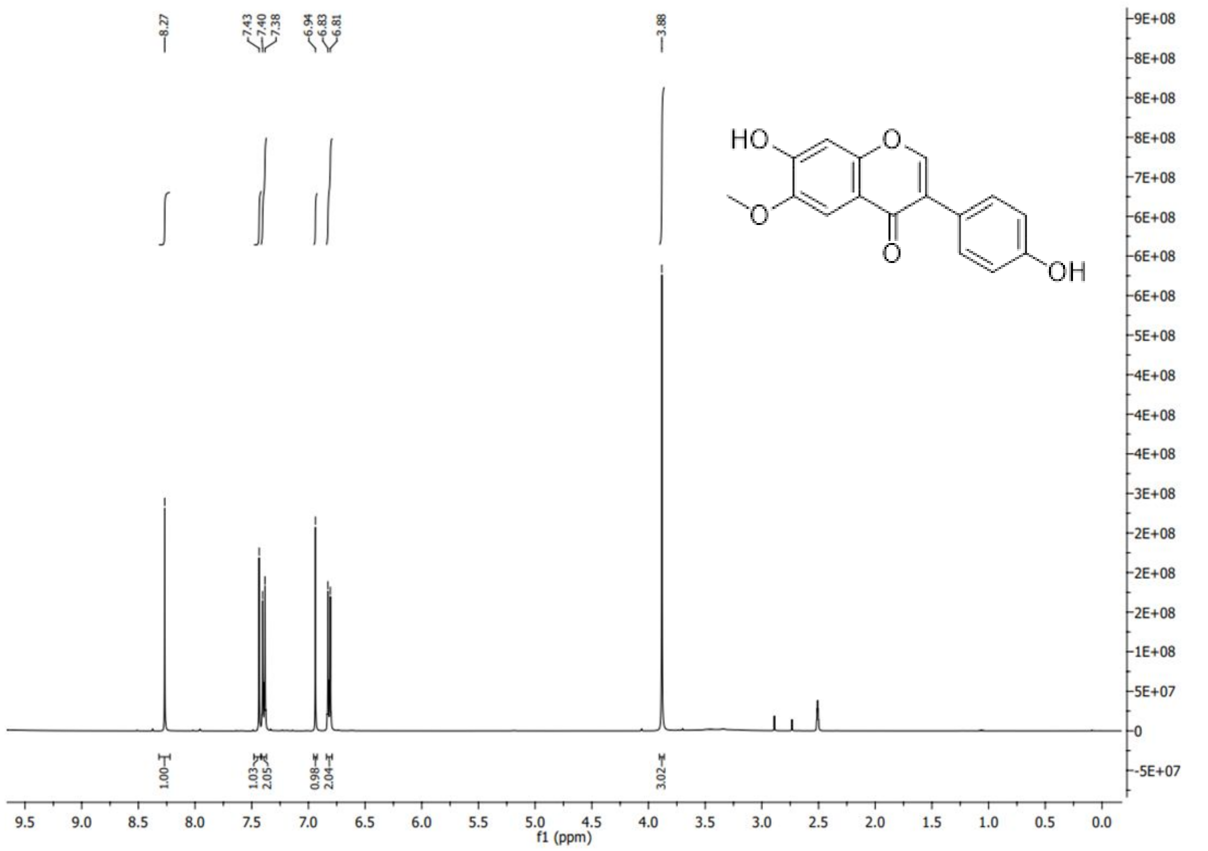

Figure S4.5: ^{1}H-NMR spectrum of glycitein.

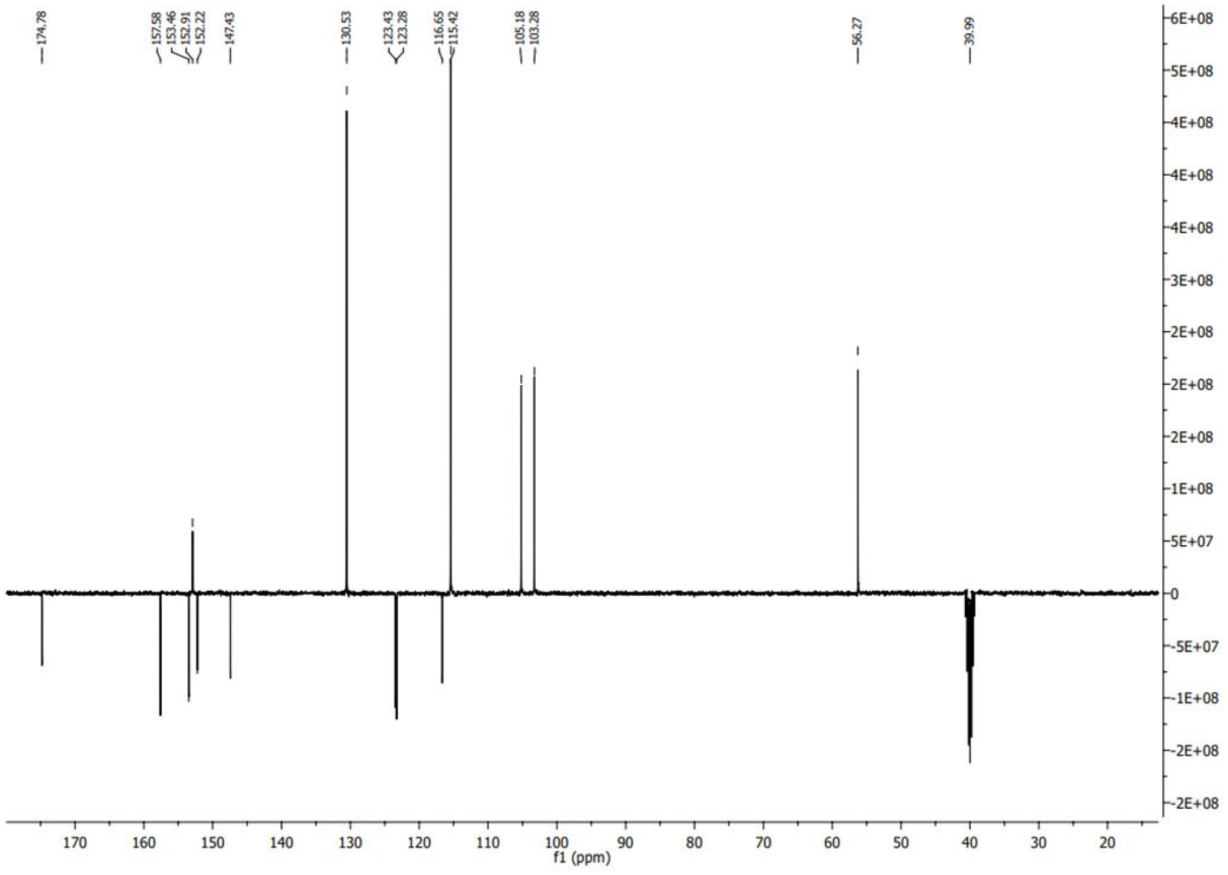

Figure S4.6: ^{13}C-NMR spectrum of glycitein.

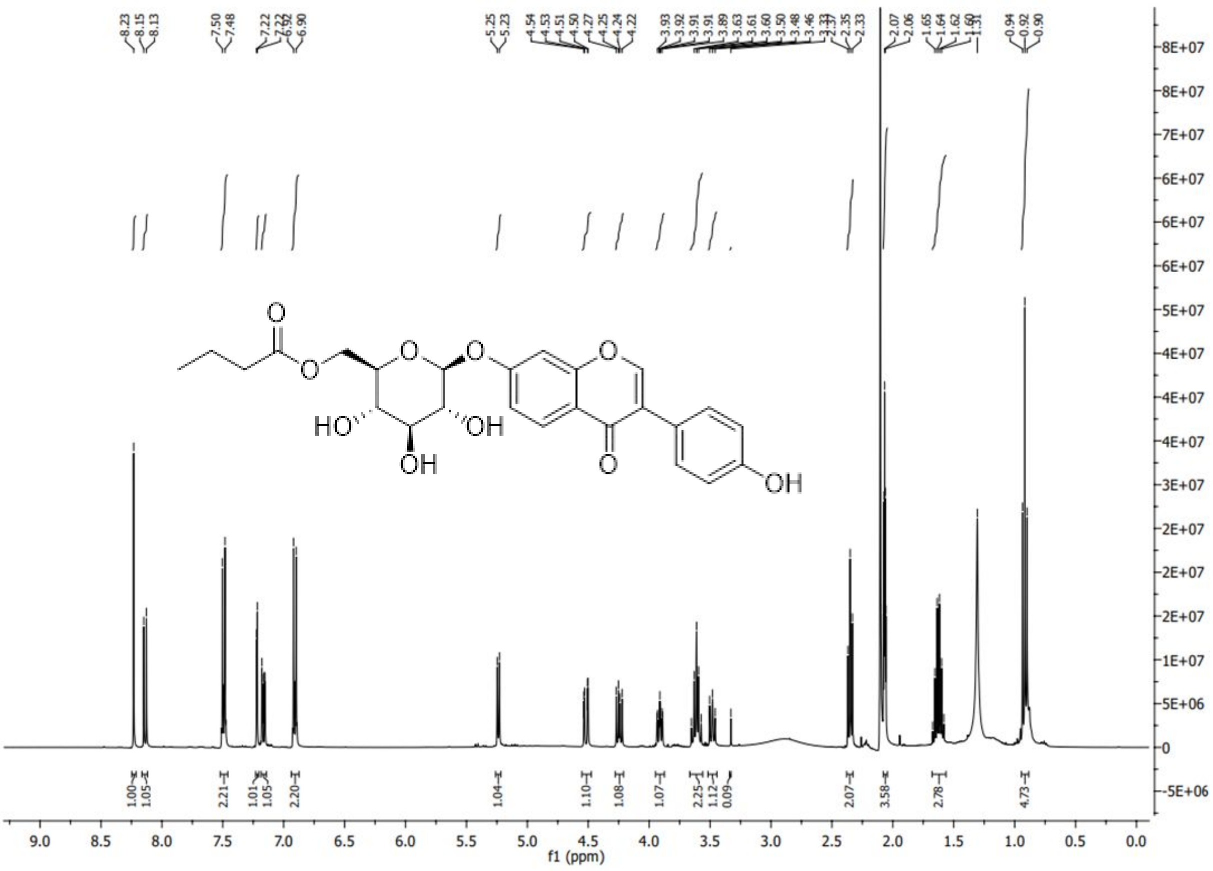

Figure S4.7: ^{1}H-NMR spectrum of 6-O-butanoildaidzin.

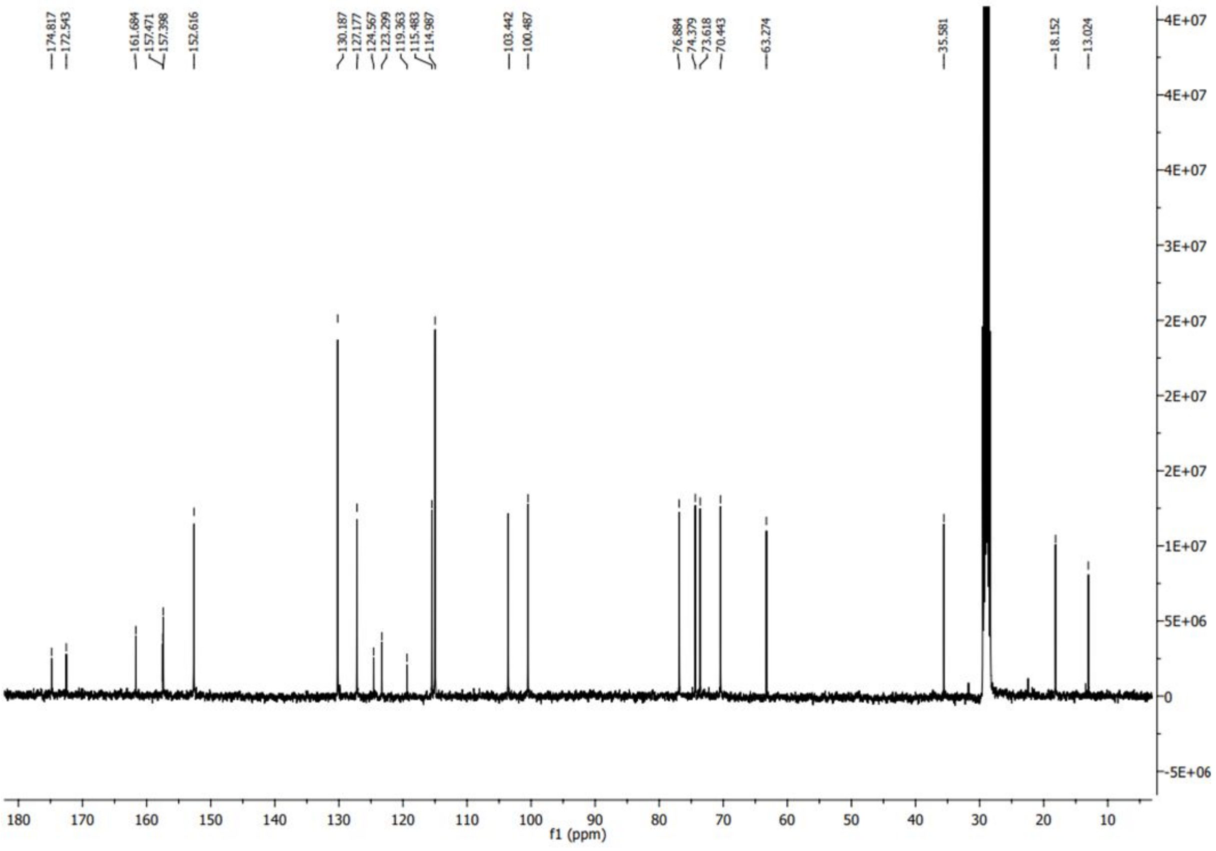

Figure S4.8: ^{13}C-NMR spectrum of 6-O-butanoildaidzin.

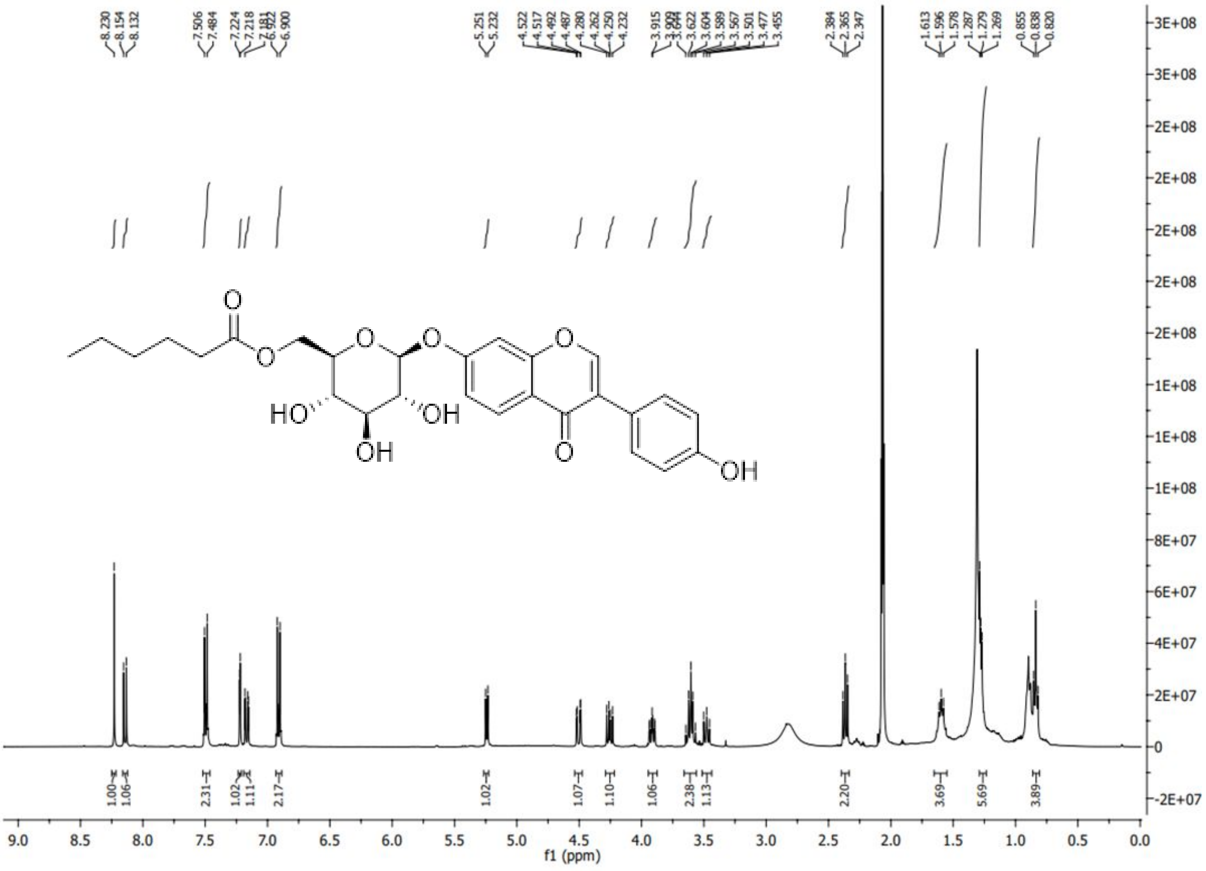

Figure S4.9: ^{1}H-NMR spectrum of 6-O-hexanoildaidzin.

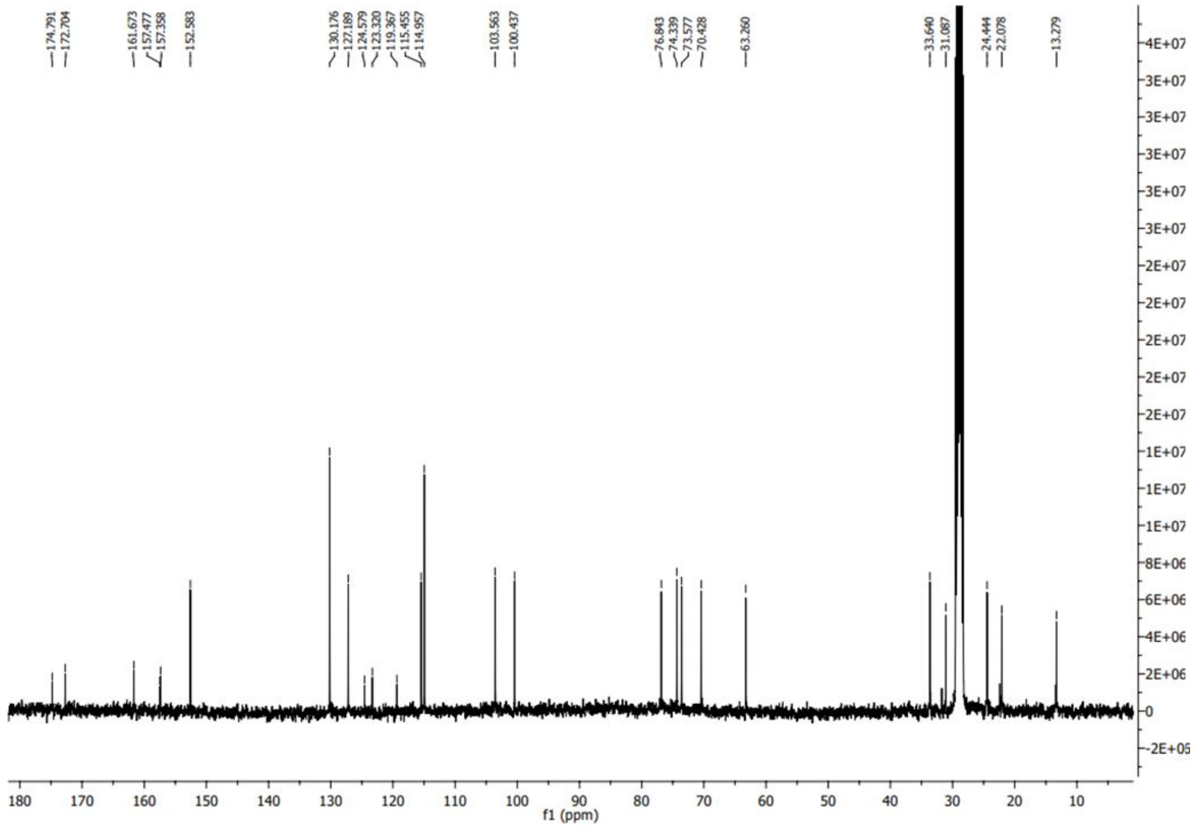

Figure S4.10: ^{13}C-NMR spectrum of 6-O-hexanoildaidzin.

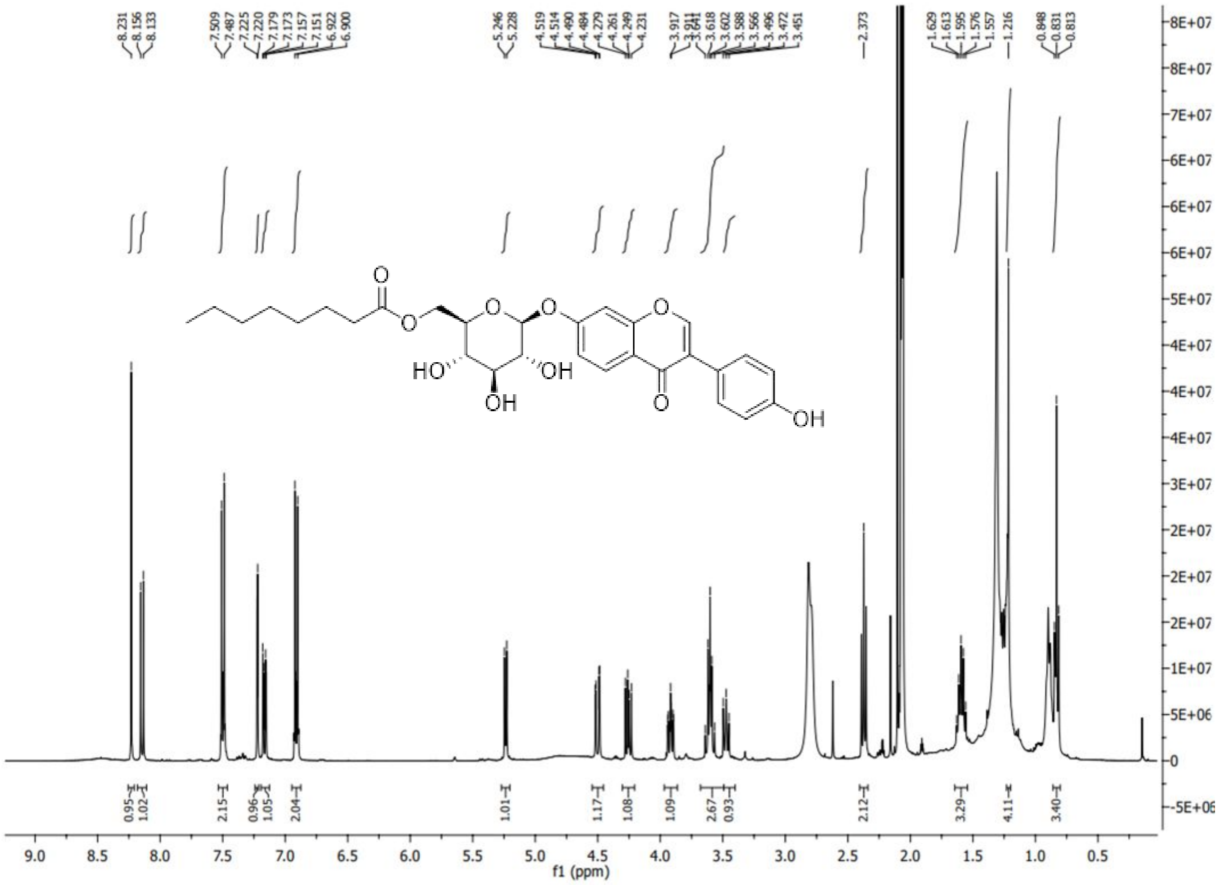

Figure S4.11: ^{1}H-NMR spectrum of 6-O-octanoildaidzin.

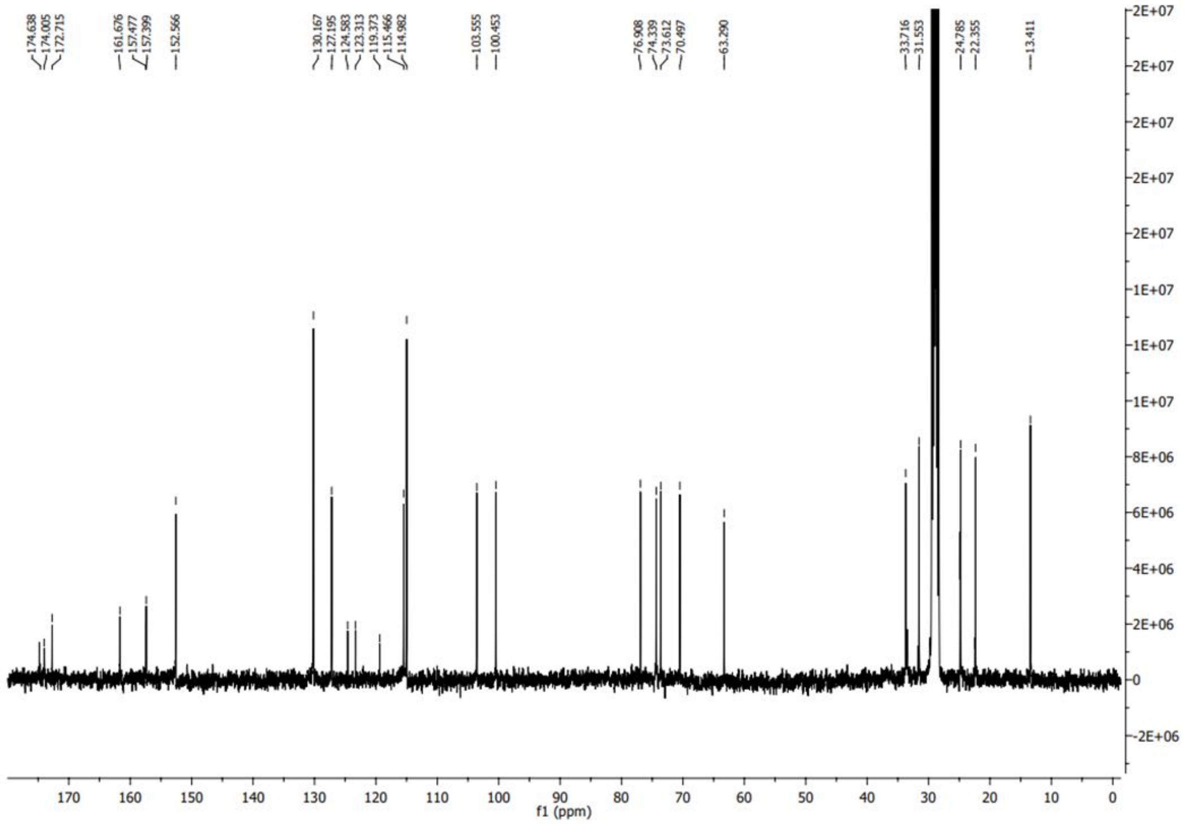

Figure S4.12: ^{13}C-NMR spectrum of 6-O-octanoildaidzin.

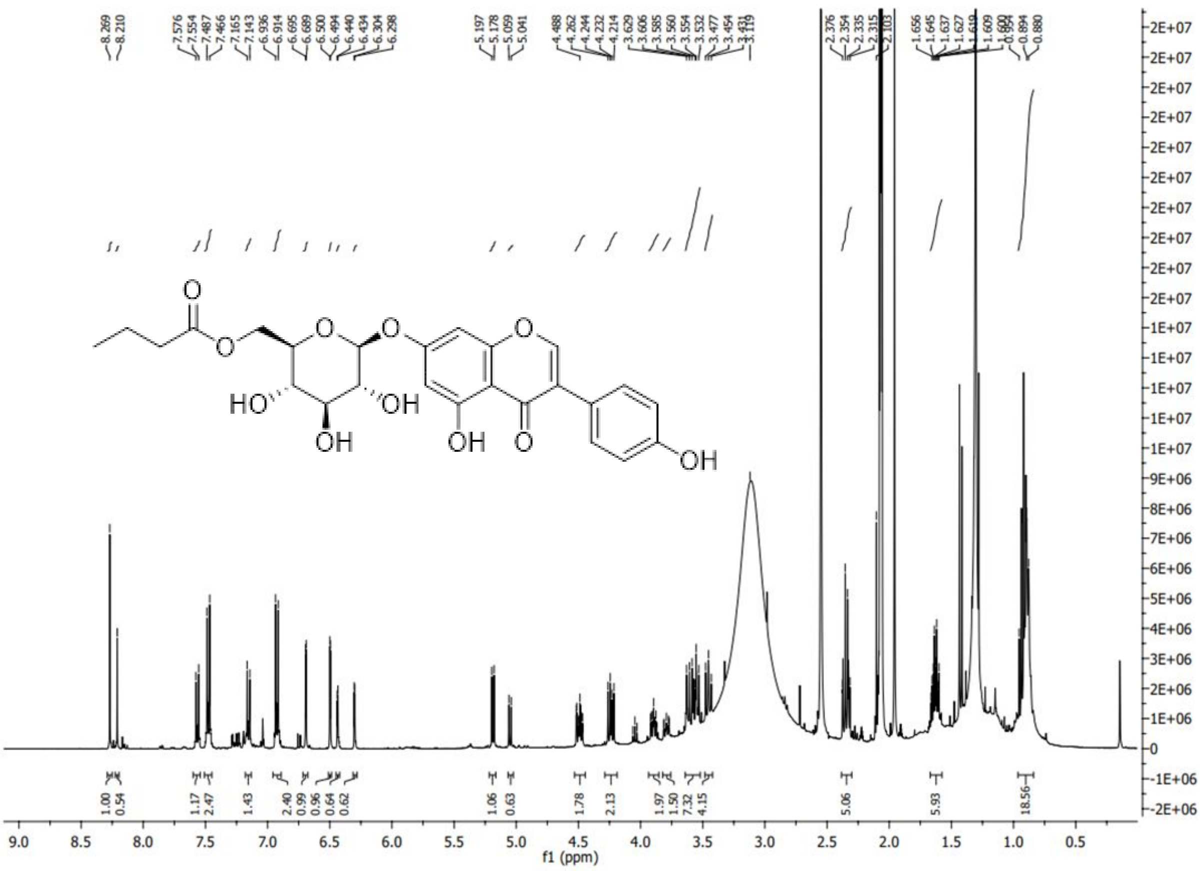

Figure S4.13: ^{1}H-NMR spectrum of 6-O-butanoilgenistin.

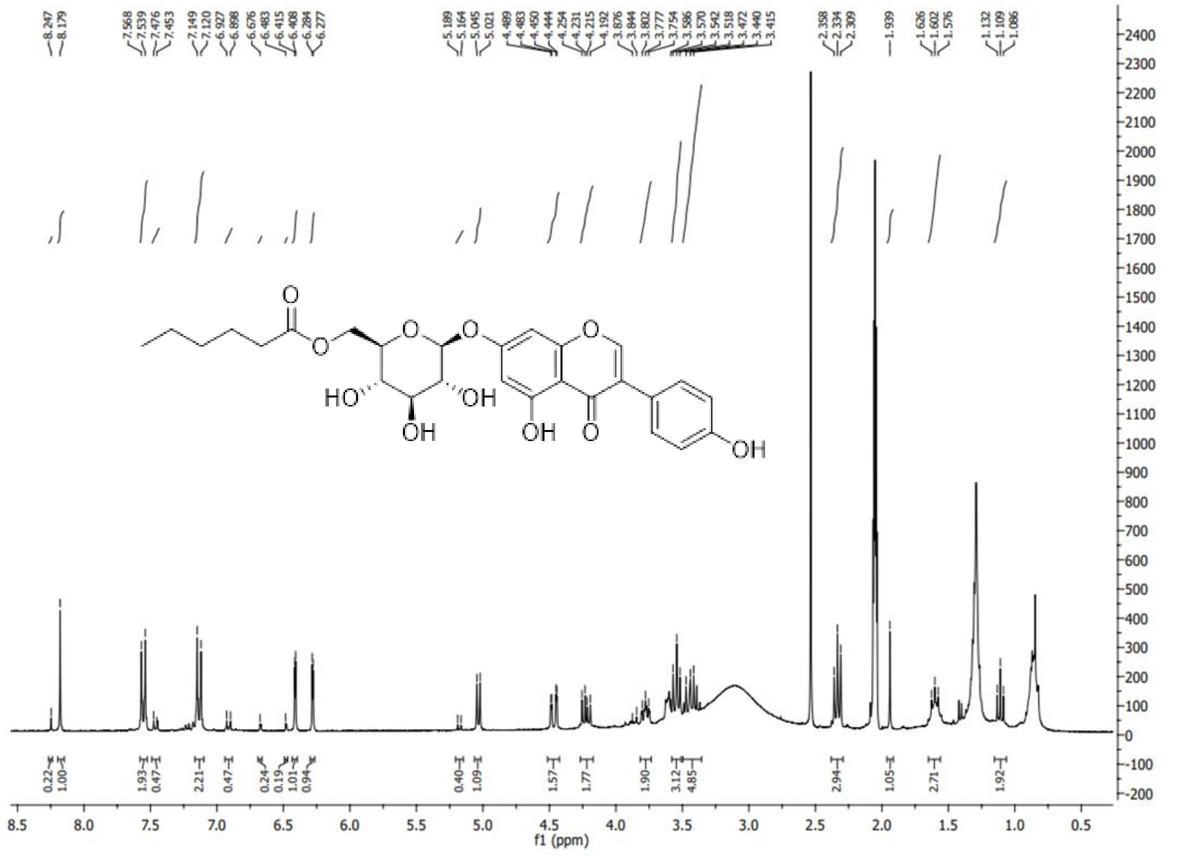

Figure S4.14: ^{1}H-NMR spectrum of 6-O-hexanoilgenistin.

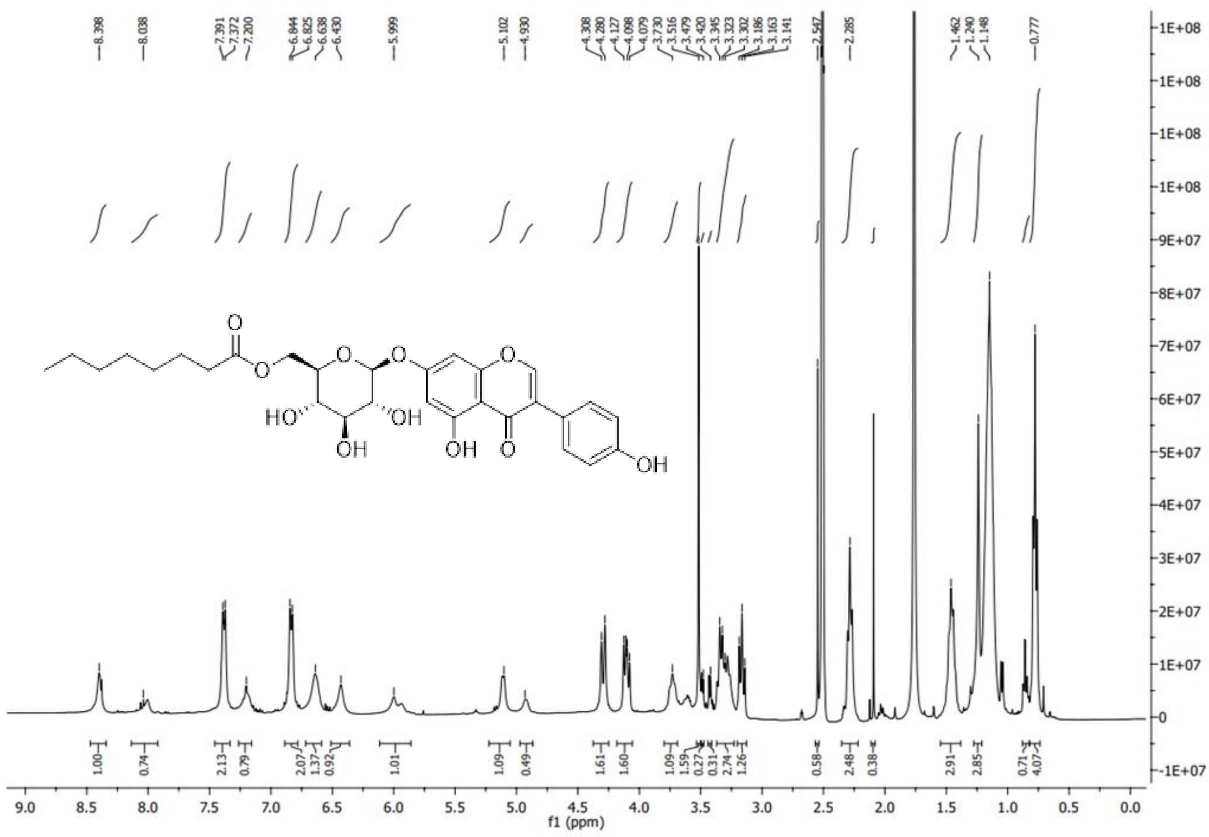

Figure S4.15: ^{1}H-NMR spectrum of 6-O-octanoilgenistin.
